# Supplementary material for: MLL-AF4 cooperates with PAF1 and FACT to drive high-density enhancer interactions in leukemia
Source: Nat Commun. 2023 Aug 25;14:5208. doi: 10.1038/s41467-023-40981-9 (PMC10457349; doi:10.1038/s41467-023-40981-9)
Supplement: Supplementary file 1 — Supplementary Information [file 41467_2023_40981_MOESM1_ESM.pdf]

MLL-AF4 cooperates with PAF1 and FACT to drive high density enhancer interactions in leukemia

Crump et al.

Supplementary Information

Supplementary Figure 1

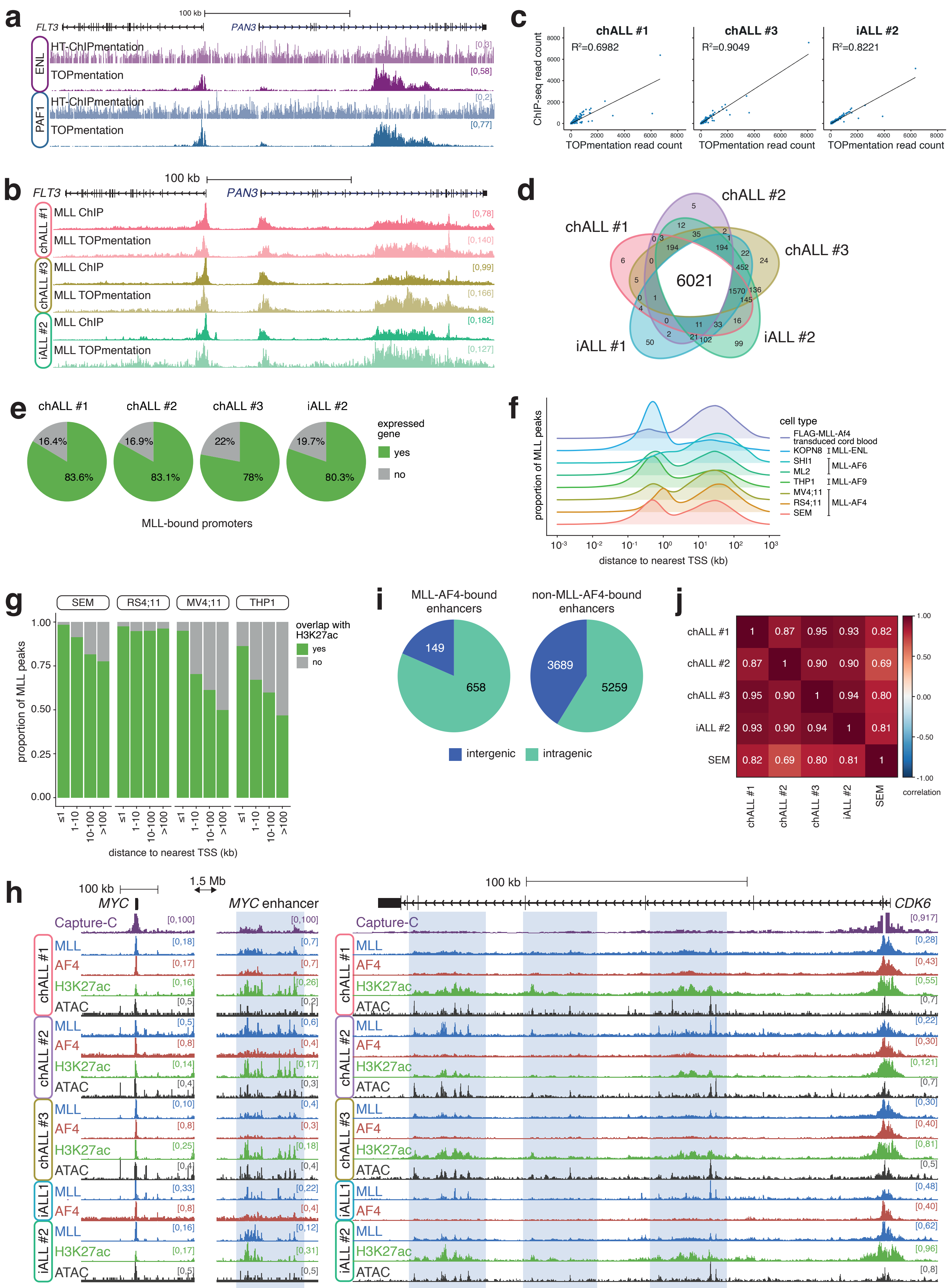

Supplementary Figure 1.

a, Comparison of ENL and PAF1 signal at the *FLT3* locus for high throughput (HT) ChIPmentation and TOPmentation in RS4;11 cells. **b**, Comparison of MLL ChIP-seq and TOPmentation at *FLT3* for the indicated MLL-AF4 ALL patients. **c**, Correlation of read counts from MLL ChIP-seq and TOPmentation at MLL peaks called using MLL ChIP-seq for the indicated MLL-AF4 ALL patients. Read counts were RPKM normalized and the R^2 value was generated using the Spearman method. **d**, Overlap of genes bound by MLL-AF4 at promoters in the indicated MLL-AF4 ALL patients. **e**, Proportion of MLL-AF4-bound gene promoters that are expressed in each patient. **f**, Distribution of MLL ChIP-seq peaks in the indicated cell lines (or distribution of FLAG tag for FLAG-MLL-Af4 transduced cells), relative to the nearest TSS. Fusion protein expressed in each cell line is indicated. **g**, Acetylation status of MLL peaks based on distance from the nearest TSS, in the indicated *MLL*r cell lines. **h**, ChIP-seq for MLL, AF4 and H3K27ac, and ATAC-seq at *MYC* (left) and *CDK6* (right) in the indicated patient samples. The *MYC* enhancer is approx. 1.7 Mb downstream of the TSS [1]. Capture-C from the TSS in SEM cells is shown. MLL-AF4-bound enhancer regions in common are highlighted in blue. **i**, Proportion of MLL-AF4-bound and non-MLL-AF4-bound enhancers located within (intragenic) or between (intergenic) genes in SEM cells. **j**, Correlation of read counts from MLL ChIP-seq from MLL-AF4 patients and SEM cells at the unified MLL-AF4-bound enhancer set.

Supplementary Figure 2

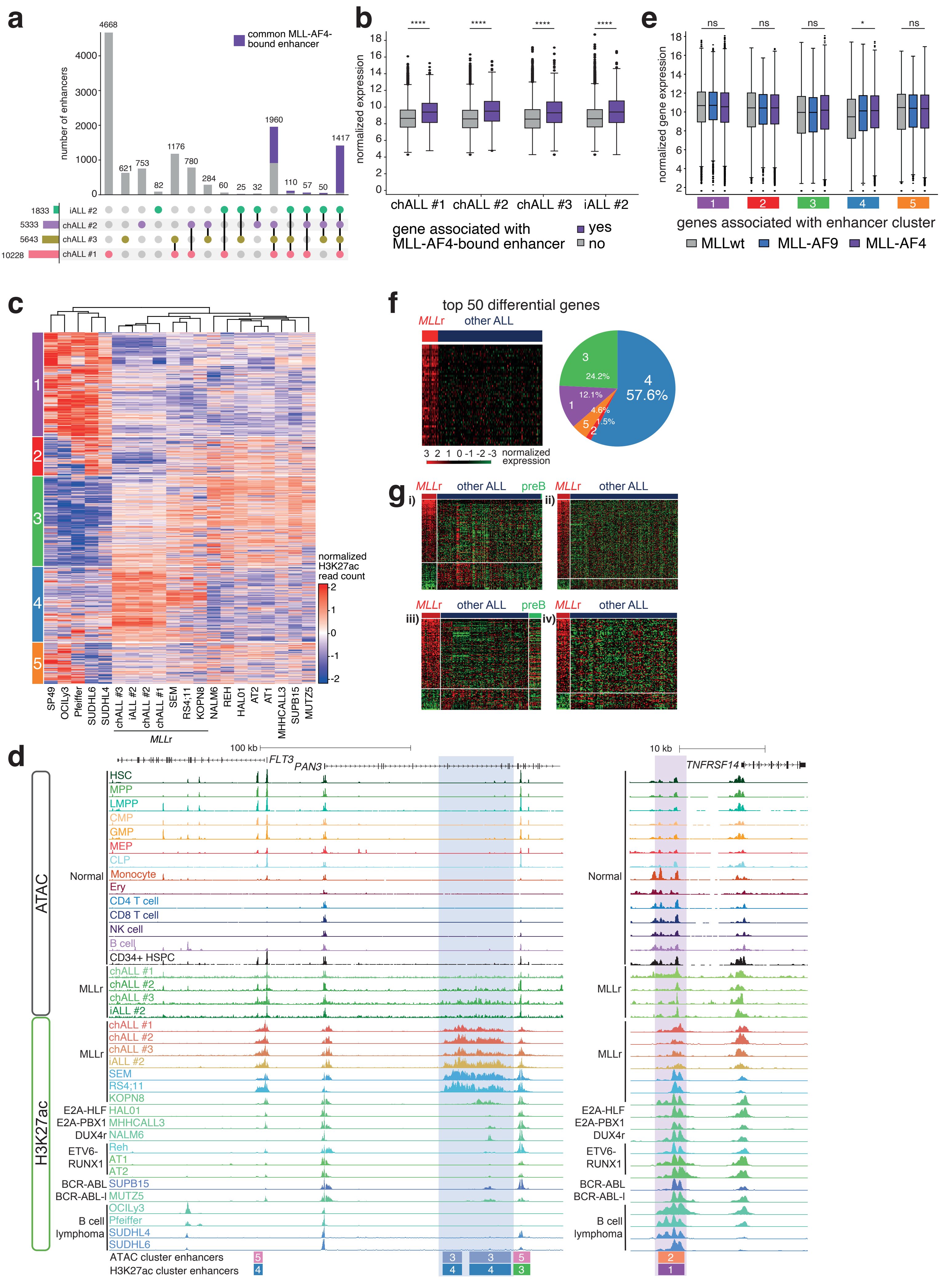

Supplementary Figure 2.

a, Overlap of enhancer usage between MLL-AF4 patients. Enhancers present in the unified MLL-AF4-bound enhancer set (bound in at least three patients) are colored purple. **b**, VST-normalized expression of genes either associated with an MLL-AF4-bound enhancer (n=973) or not (n=8942) for the indicated patient samples, n=1 for each patient. ****p<0.0001; p=1.59x10^{-34} (chALL #1), 2.45x10^{-52} (chALL #2), 3.09x10^{-32} (chALL #3), 4.73x10^{-36} (iALL #2) (two-sided Mann-Whitney U test). Midline shows median, with upper and lower hinges showing 25th and 75th percentile, respectively. Upper and lower hinges extend to the largest and smallest datapoints within 1.5 times the interquartile range of either hinge. **c**, Clustered heatmap of H3K27ac levels at unified MLL-AF4-bound enhancers in MLL-AF4 patients, *MLL*r and *MLL* wild-type ALL cell lines [2]. **d**, ATAC-seq and H3K27ac ChIP-seq at the *FLT3* and *TNFRSF14* loci in adult blood cell types [3], MLL-AF4 patients and ALL cell lines [2]. Primary translocations are indicated. The enhancer within *PAN3* in MLL-AF4 ALL cells is highlighted in blue. **e**, VST-normalized expression of genes associated with each cluster of MLL-AF4-bound enhancers indicated in (c), for MLL wild-type (n=10), MLL-AF4 (n=35) and MLL-AF9 (n=5) patient samples [4]. * p.adj<0.05; ns: no significant difference; for MLLwt vs MLL-AF4, p.adj=0.90 (cluster 1), p.adj=0.90 (cluster 2), p.adj=0.66 (cluster 3), p.adj=0.03 (cluster 4), p.adj=0.90 (cluster 5) (two-sided Mann-Whitney U test, corrected for multiple testing using the Benjamini-Hochberg method). Midline shows median, with upper and lower hinges showing 25th and 75th percentile, respectively. Upper and lower hinges extend to the largest and smallest datapoints within 1.5 times the interquartile range of either hinge. **f**, Left panel: Microarray expression analysis (ECOG E2993 [5]) of all genes associated with an MLL-AF4 enhancer. The 50 most differential genes between *MLL*r and other ALL patients are shown. Right panel: Proportion of the 50 most differential genes between *MLL*r and other ALL samples associated with each enhancer cluster indicated in (c). **g**, Microarray expression analysis of genes in four different patient datasets: i) ECOG E2993 [5]; ii) COG P9906 [6]; iii) St Jude 2003 [7] and iv) St Jude 2013 [8]. Genes used in the analysis were taken from cluster 4 (MLL-AF4 specific) in (c).

Supplementary Figure 3

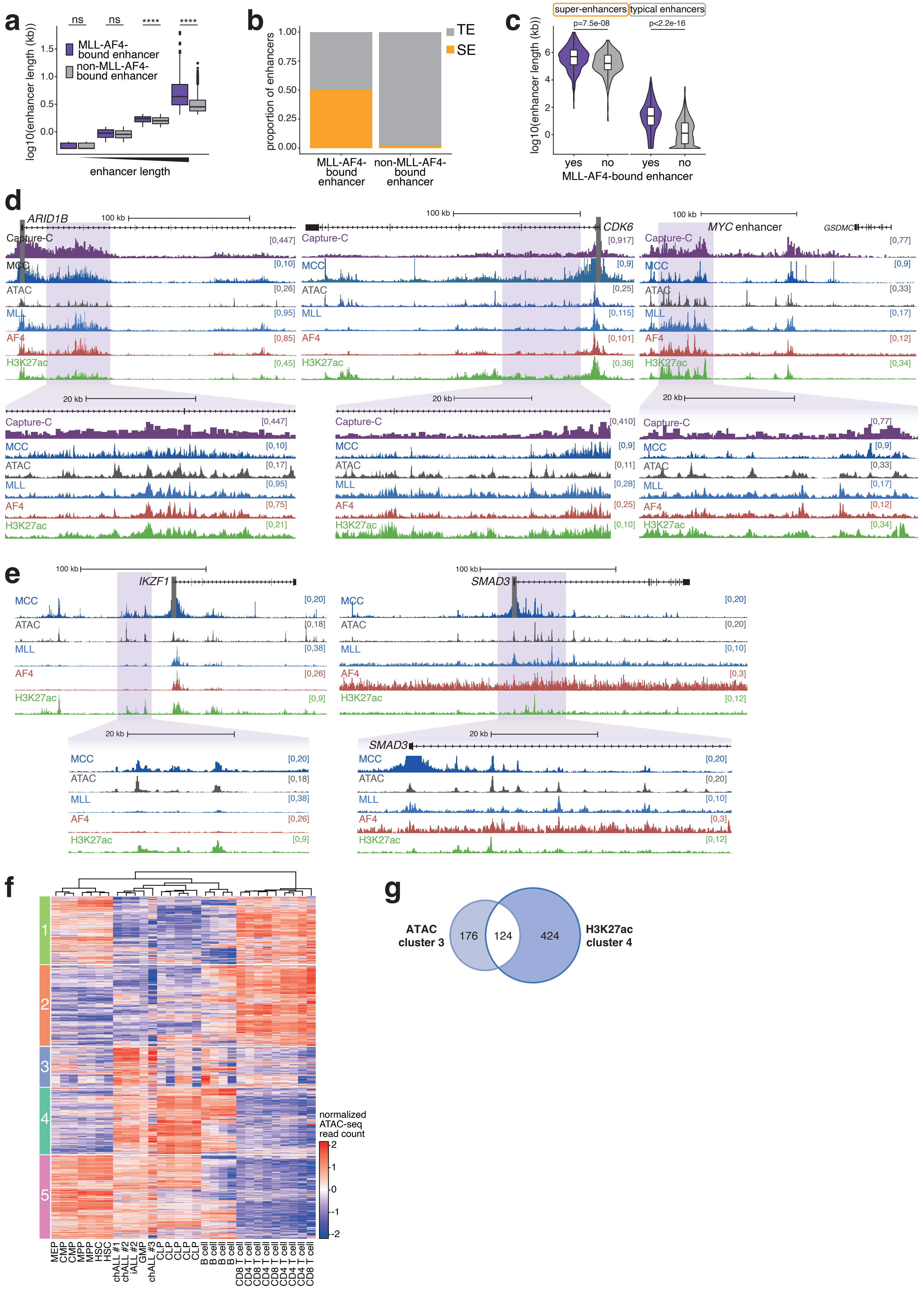

Supplementary Figure 3.

a, Distribution of the length of enhancers bound (n=807) or not bound (n=8948) by MLL-AF4 in SEM cells, stratified into quartiles by enhancer length (irrespective of MLL-AF4 binding status). Midline shows median, with upper and lower hinges showing 25th and 75th percentile, respectively. **** p<0.0001; ns: no significant difference; p=0.9757 (Q1), 0.0669 (Q2), 4.095×10^{-5} (Q3), $p<2.2 \times 10^{-16}$ (Q4) (two-sided Wilcoxon rank sum test). Upper and lower hinges extend to the largest and smallest datapoints within 1.5 times the interquartile range of either hinge. **b**, Proportion of MLL-AF4-bound and unbound enhancers in SEM cells that are classified as super-enhancers. **c**, Distribution of the length of super- and typical enhancers bound (n=547 SEs, 533 TEs) or not bound (n=177 SEs, 7970 TEs) by MLL-AF4 in SEM cells. Statistical significance calculated using a two-sided Mann-Whitney U test; $p=7.5 \times 10^{-8}$ (SEs), $p<2.2 \times 10^{-16}$ (TEs). Midline shows median, with upper and lower hinges showing 25th and 75th percentile, respectively. Upper and lower hinges extend to the largest and smallest datapoints within 1.5 times the interquartile range of either hinge. **d**, Capture-C, Micro-Capture-C (MCC), ATAC-seq and ChIP-seq for MLL, AF4 and H3K27ac at the *ARID1B*, *CDK6* and *MYC* enhancer loci in SEM cells. Enhancer regions are highlighted in purple. Capture-C and MCC traces scaled to emphasize distal interactions. **e**, Micro-Capture-C (MCC), ATAC-seq and ChIP-seq for MLL, AF4 and H3K27ac at the *IKZF3* and *SMAD3* loci in SEM cells. Enhancer regions are highlighted in purple. MCC traces scaled to emphasize distal interactions. **f**, Clustered heatmap of ATAC-seq levels at unified MLL-AF4-bound enhancers in MLL-AF4 patients and adult blood cell types [3]. **g**, Overlap of MLL-AF4-bound enhancers found within cluster 3 of adult blood cell ATAC analysis (Supplementary Fig. 2c) and cluster 4 of ALL cell H3K27ac analysis (f).

Supplementary Figure 4

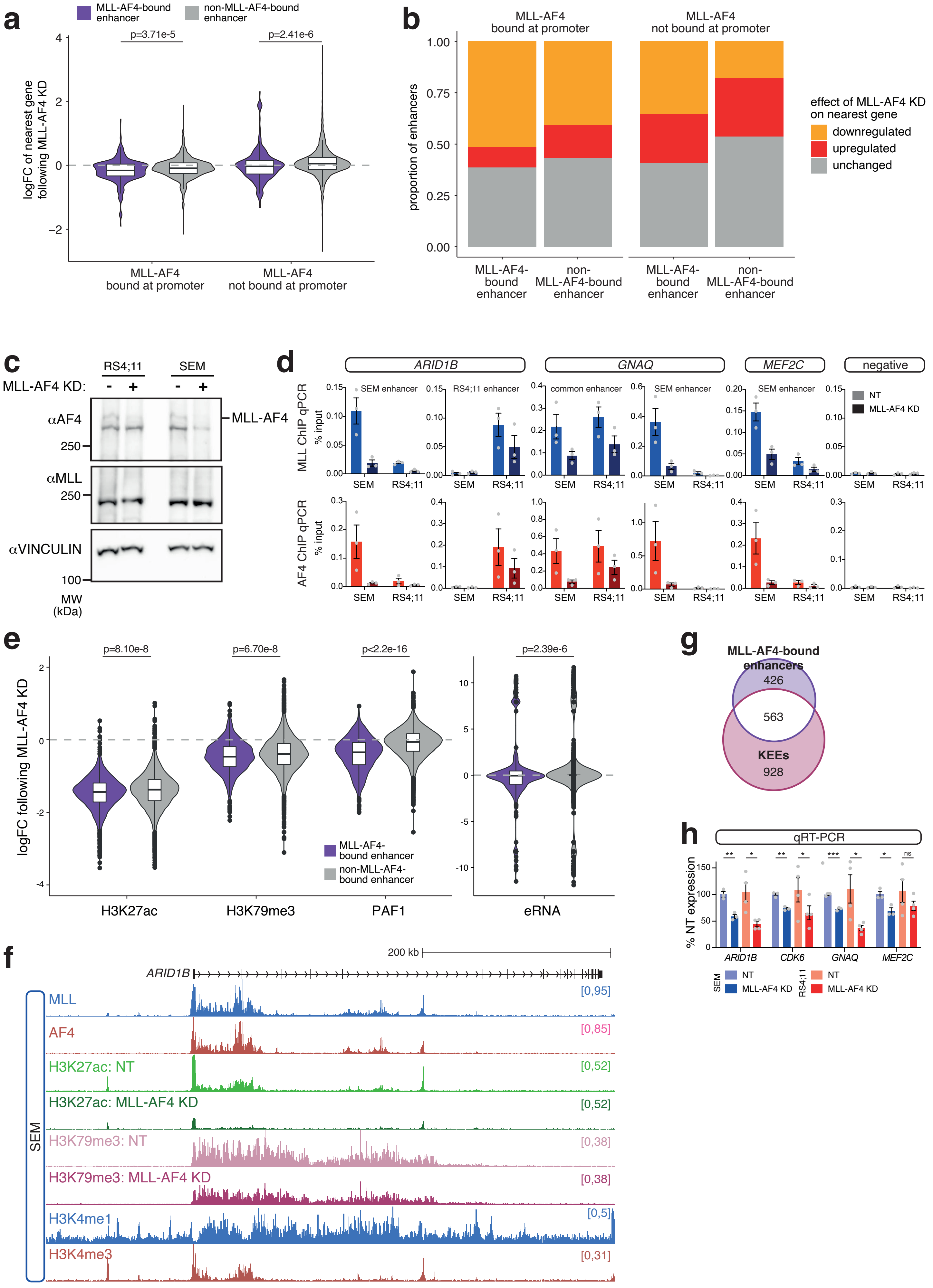

Supplementary Figure 4.

a, Change in gene expression following 96h knockdown of MLL-AF4 in SEM cells, separated by the presence or absence of MLL-AF4 within 5 kb of the TSS, for genes associated with an MLL-AF4-bound enhancer (n=807) and genes associated with an enhancer not bound by MLL-AF4 (n=8948), mean of three independent experiments. Statistical significance calculated using a two-sided Mann–Whitney U test. Midline shows median, with upper and lower hinges showing 25th and 75th percentile, respectively. Upper and lower hinges extend to the largest and smallest datapoints within 1.5 times the interquartile range of either hinge. **b**, Proportion of enhancers associated with genes displaying each transcriptional response to MLL-AF4 knockdown, separated by the presence or absence of MLL-AF4 within 5 kb of the TSS. **c**, Western blot for AF4 (detecting MLL-AF4) and wild-type MLL in control (non-targeting; -) and 96h MLL-AF4 knockdown (+) RS4;11 and SEM cells. VINCULIN is shown as a loading control. Blots are representative of three replicates. Source data are provided at the end of this document. **d**, ChIP-qPCR for MLL and AF4 at the indicated enhancer regions in SEM and RS4;11 cells, under control (NT) and MLL-AF4 knockdown conditions at 96h. Data are represented as mean ± SEM, n=3 independent experiments. Source data are provided as a Source Data file. **e**, Log2 fold-change in levels of H3K27ac, H3K79me3, PAF1 and RNA transcription at MLL-AF4-bound (n=807) and unbound (n=8948) enhancers following 96h MLL-AF4 knockdown in SEM cells. Mean of three independent experiments for eRNA. Statistical significance calculated using a two-sided Wilcoxon rank sum test; $p=8.10x10^{-8}$ (H3K27ac), $p=6.70x10^{-8}$ (H3K79me3), $p<2.2x10^{-16}$ (PAF1), $p=2.39x10^{-6}$ (eRNA). Midline shows median, with upper and lower hinges showing 25th and 75th percentile, respectively. Upper and lower hinges extend to the largest and smallest datapoints within 1.5 times the interquartile range of either hinge. **f**, Reference-normalized ChIP-seq for H3K27ac and H3K79me3 at *ARID1B* in SEM cells under control (NT) and MLL-AF4 96h knockdown conditions. ChIP-seq for MLL, AF4, H3K4me1 and H3K4me3 is shown for context. **g**, Overlap of MLL-AF4-bound enhancers and KEEs in SEM cells. **h**, qRT-PCR analysis of gene expression in SEM and RS4;11 cells following MLL-AF4 knockdown. Data are represented as mean ± SEM, n=3 independent experiments. ns: non-significant; * p<0.05; ** p<0.01; *** p<0.001; SEM p-values: 0.0032 (*ARID1B*), 0.0013 (*CDK6*), 0.0003 (*GNAQ*), 0.0198, (*MEF2C*), RS4;11 p-values: 0.0152 (*ARID1B*), 0.0407 (*CDK6*), 0.0347 (*GNAQ*), 0.2786 (*MEF2C*) (two-sided unpaired t-test) (two-sided unpaired t-test). Source data are provided as a Source Data file.

Supplementary Figure 5

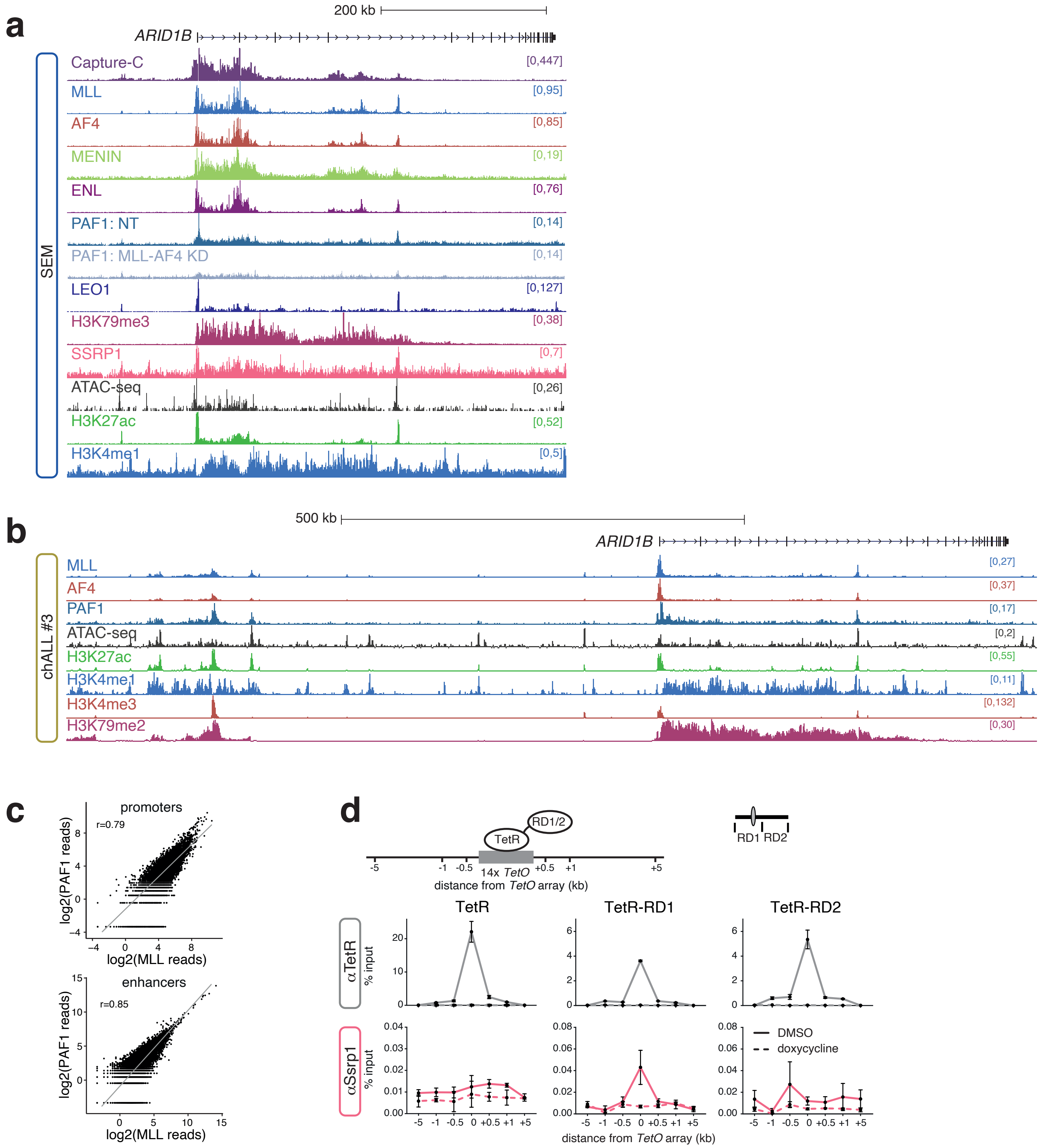

Supplementary Figure 5.

a, ChIP-seq, ATAC-seq and Capture-C at the *ARID1B* locus in SEM cells. Reference-normalized ChIP-seq for PAF1 in SEM cells under control (NT) and MLL-AF4 knockdown conditions. The Capture-C viewpoint is the *ARID1B* TSS. **b**, TOPmentation and ATAC-seq at the *ARID1B* locus in chALL patient #3. **c**, Correlation of MLL and PAF1 TOPmentation signal at promoters and enhancers in chALL patient #3. **d**, ChIP-qPCR for TetR (FS2) and Ssrp1 at the *TetO* array inserted into mESCs expressing TetR (not fused to another protein), or TetR fused to the RD1 or RD2 fragments of the MLL CXXC domain. Dashed line shows ChIP-qPCR in cells treated with doxycycline for 6h. Data are represented as mean ± SEM, n≥2. Source data are provided as a Source Data file.

Supplementary Figure 6

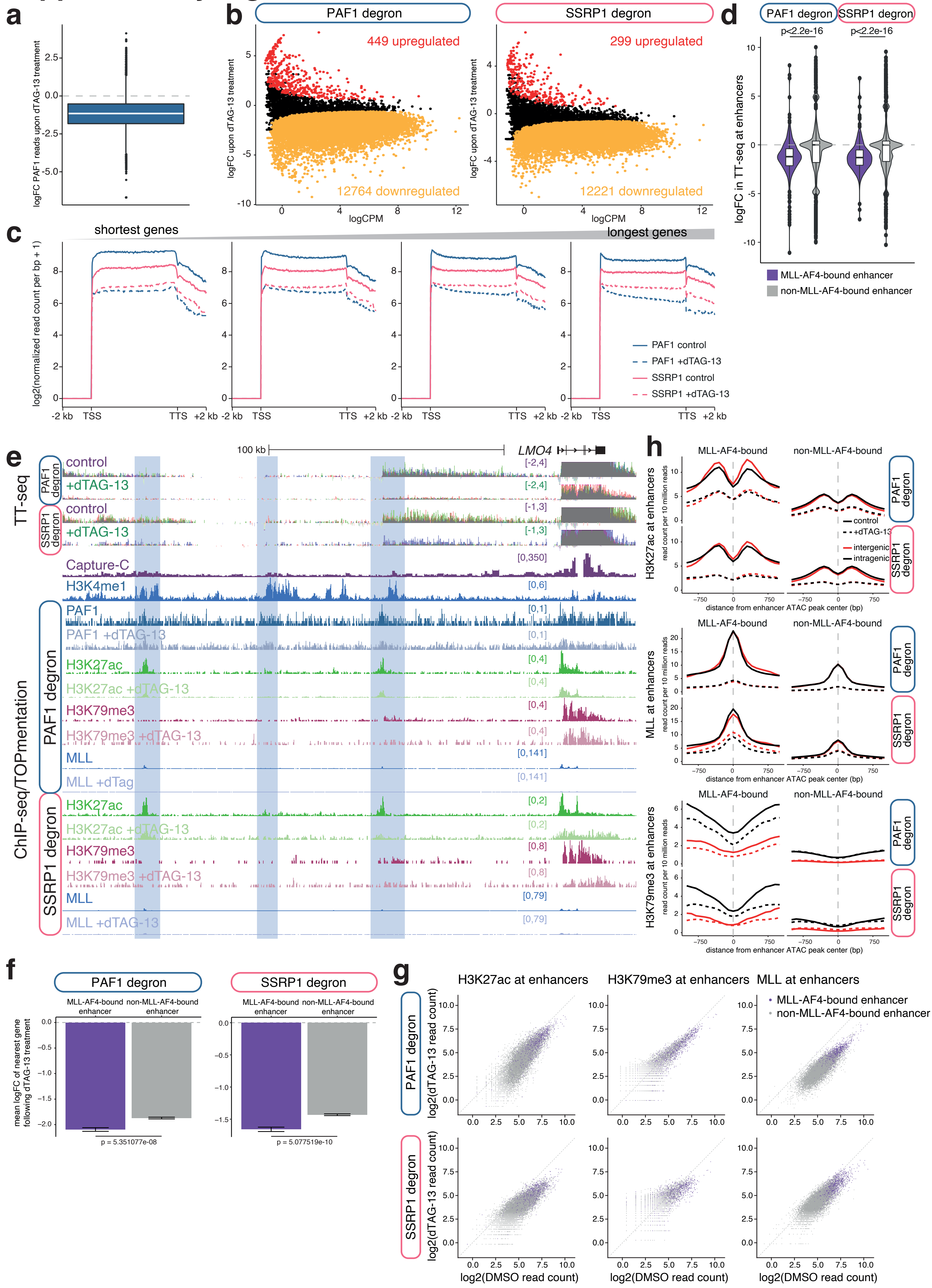

Supplementary Figure 6.

a, Difference in TOPmentation PAF1 reads at PAF1 peaks in untreated or 24h dTAG-13-treated PAF1 degron cells, n=1. Midline shows median, with upper and lower hinges showing 25th and 75th percentile, respectively. Upper and lower hinges extend to the largest and smallest datapoints within 1.5 times the interquartile range of either hinge. **b**, MA plots of TT-seq data showing the effect of 24h dTAG-13 treatment on gene expression in PAF1 degron or SSRP1 degron SEM cell lines; FDR<0.05, n=3 independent experiments. **c**, Metagene profiles of TT-seq levels across gene bodies in PAF1 degron or SSRP1 degron cell lines, stratified into quartiles by gene length. **d**, Log2 fold-change in levels of RNA transcription at MLL-AF4-bound (n=807) and unbound (n=8948) enhancers following 24h dTAG-13 treatment of PAF1 degron or SSRP1 degron SEM cell lines, mean of three independent experiments. Statistical significance calculated using a two-sided Mann–Whitney U test; $p<2.2x10^{-16}$ for both the PAF1 and SSRP1 degron. Midline shows median, with upper and lower hinges showing 25th and 75th percentile, respectively. Upper and lower hinges extend to the largest and smallest datapoints within 1.5 times the interquartile range of either hinge. **e**, TT-seq, reference-normalized ChIP-seq and TOPmentation at the *LMO4* locus in PAF1 degron and SSRP1 degron SEM cells, with or without the addition of dTAG-13 for 24h. Capture-C and H3K4me1 ChIP-seq from control SEM cells are shown for reference. The Capture-C viewpoint is the *LMO4* TSS. **f**, Mean logFC of transcription at genes associated with an MLL-AF4-bound (n=807) or -unbound (n=8948) enhancer, following 24h dTAG-13 treatment of PAF1 degron or SSRP1 degron cell lines, n=3 independent experiments. Statistical significance calculated using a two-sided Wilcoxon rank sum test; $p=5.35x10^{-8}$ (PAF1 degron), $5.08x10^{-10}$ (SSRP1 degron). Midline shows median, with upper and lower hinges showing 25th and 75th percentile, respectively. Upper and lower hinges extend to the largest and smallest datapoints within 1.5 times the interquartile range of either hinge. **g**, Levels of H3K27ac, H3K79me3 and MLL at MLL-AF4-bound (purple) and non-MLL-AF4-bound (gray) enhancers following 24h dTAG-13 treatment of PAF1 degron or SSRP1 degron SEM cell lines. **h**, Mean distribution of H3K27ac, MLL and H3K79me3 at intergenic (red) and intragenic (black) MLL-AF4-bound and non-MLL-AF4-bound enhancers, in PAF1 degron or SSRP1 degron cell lines under control (untreated; solid line) and dTAG-13-treated (dashed line) conditions.

Supplementary Figure 7

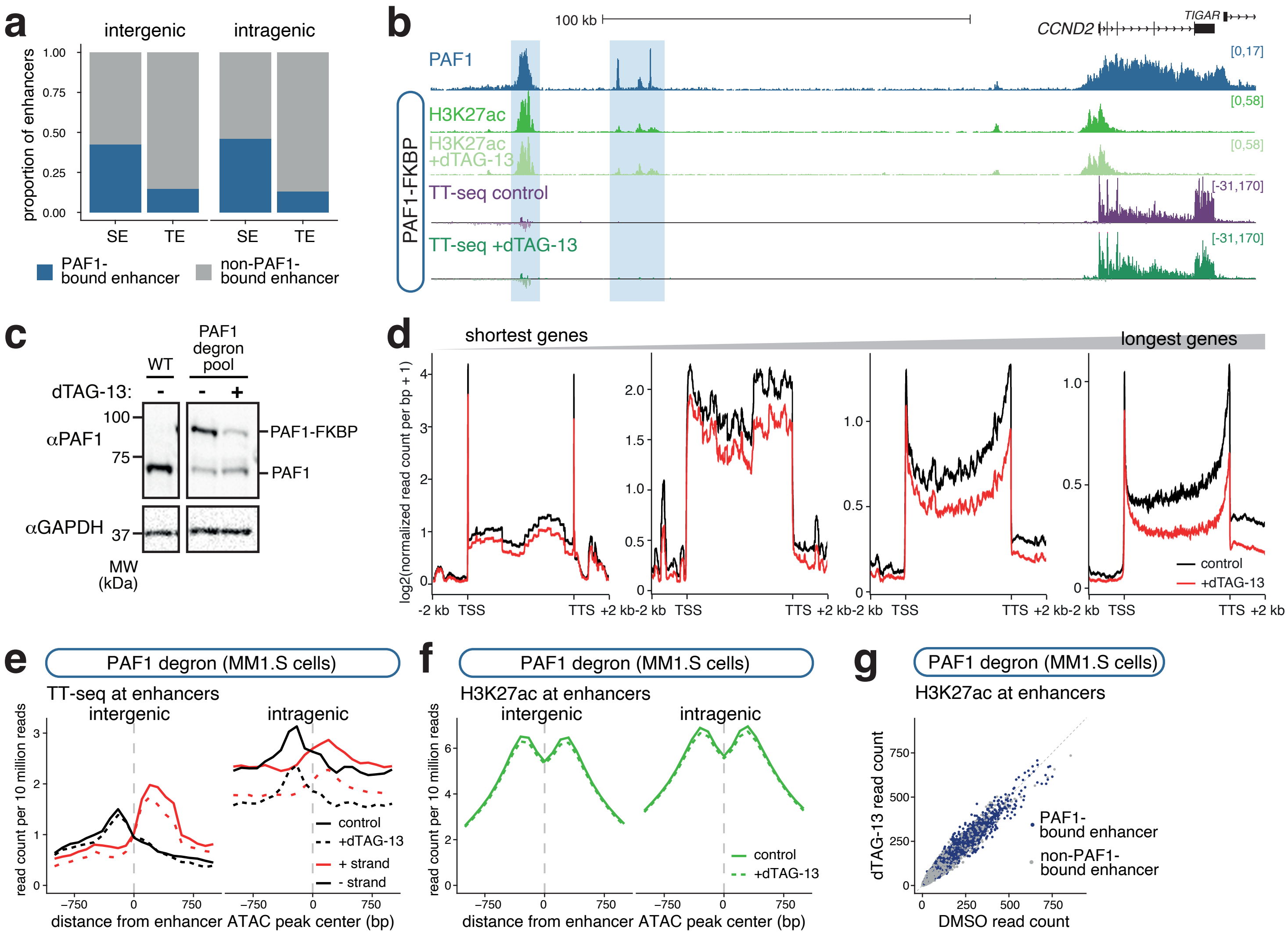

Supplementary Figure 7.

a, Proportion of super- (SE) and typical (TE) enhancers bound by PAF1 in MM1.S cells. **b**, PAF1 ChIP-seq, reference-normalized H3K27ac ChIP-seq and TT-seq at the *CCND2* locus in PAF1 degron MM1.S cells, with or without the addition of dTAG-13 for 24h. Enhancers are highlighted in blue. **c**, Western blot for PAF1 in wild-type (WT) and a pool of PAF1 degron MM1.S cells, with (+) or without (-) addition of 0.5 μM dTAG-13 for 24h. Bands representing wild-type and FKBP12 ^{F36V}-tagged PAF1 are indicated. Blots are representative of three replicates. Source data are provided at the end of this document. **d**, Metagene profiles of TT-seq levels across gene bodies in PAF1 degron MM1.S cells under control (untreated) and 24h dTAG-13-treated conditions, stratified into quartiles by gene length. **e**, Mean distribution of strand-specific TT-seq (eRNA) levels at inter- and intragenic enhancers, in PAF1 degron MM1.S cells under control (untreated) and 24h dTAG-13-treated conditions. **f**, Mean distribution of H3K27ac at inter- and intragenic enhancers, in PAF1 degron MM1.S cells under control (untreated) and 24h dTAG-13-treated conditions. **g**, H3K27ac levels at PAF1-bound and -unbound enhancers, in PAF1 degron MM1.S cells under control (untreated) and 24h dTAG-13-treated conditions.

Supplementary Figure 8

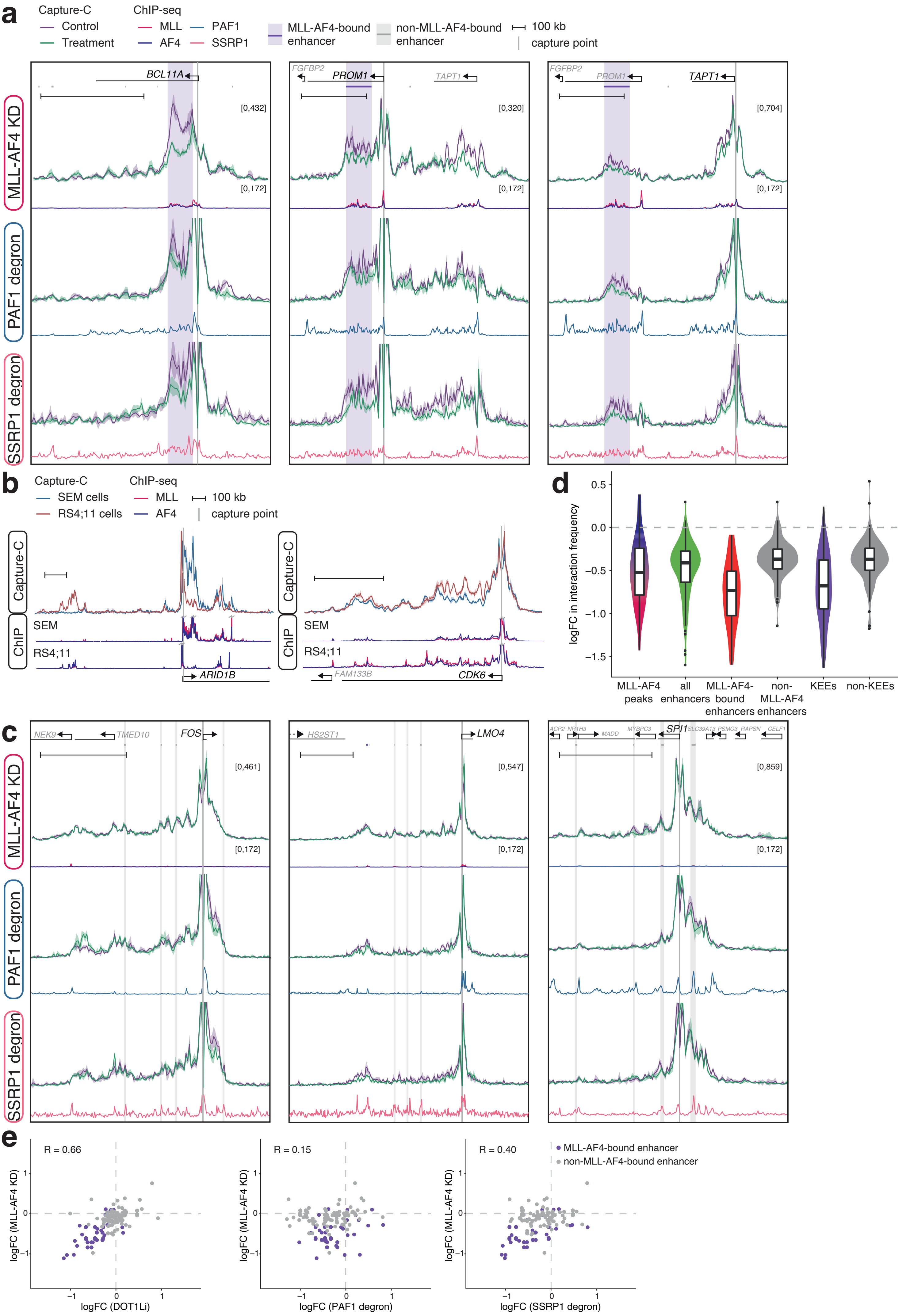

Supplementary Figure 8.

a, Capture-C from the promoters of *BCL11A*, *PROM1* and *TAPT1* in SEM cells under control (purple) and 96h MLL-AF4 knockdown (green) conditions (*upper*) or in PAF1 degron or SSRP1 degron cell lines under control (purple) and 24h dTAG-13-treated (green) conditions. Capture-C traces are scaled to emphasize distal interactions. Lines represent mean, shading represents ± SEM, n=3 independent experiments. ChIP-seq traces for MLL, AF4, PAF1 and SSRP1 are shown, along with bioinformatically-annotated MLL-AF4-bound (purple bars) and -unbound (gray bars) enhancers. **b**, Overlayed Capture-C from the promoters of *ARID1B* and *CDK6* and ChIP-seq for MLL and AF4, in SEM and RS4;11 cells. Lines represent mean, shading represents ± SEM, n=3 independent experiments. ChIP-seq tracks are scaled to emphasize differences in enhancer binding; gray bolts indicate where signal extends beyond the axis. **c**, Capture-C from the promoters of *FOS*, *LMO4* and *SPI1* in SEM cells under control (green) and 96h MLL-AF4 knockdown (purple) conditions (*upper*) or in PAF1 degron or SSRP1 degron cell lines under control (green) and 24h dTAG-13-treated (purple) conditions. Capture-C traces are scaled to emphasize distal interactions. Lines represent mean, shading represents ± SEM, n=3 independent experiments. ChIP-seq traces for MLL, AF4, PAF1 and SSRP1 are shown, along with bioinformatically-assigned MLL-AF4-bound (purple bars) and -unbound (gray bars) enhancers. **d**, Change in Capture-C interaction frequency between promoters and the indicated genomic loci, following 96h MLL-AF4 knockdown, mean of three independent experiments. Midline shows median, with upper and lower hinges showing 25th and 75th percentile, respectively. Upper and lower hinges extend to the largest and smallest datapoints within 1.5 times the interquartile range of either hinge. **e**, Change in Capture-C interaction frequency between promoters and MLL-AF4-bound (purple) and -unbound (gray) enhancers following the indicated treatments. Capture-C data under 7d DOT1Li conditions was previously published [9].

Supplementary Figure 9

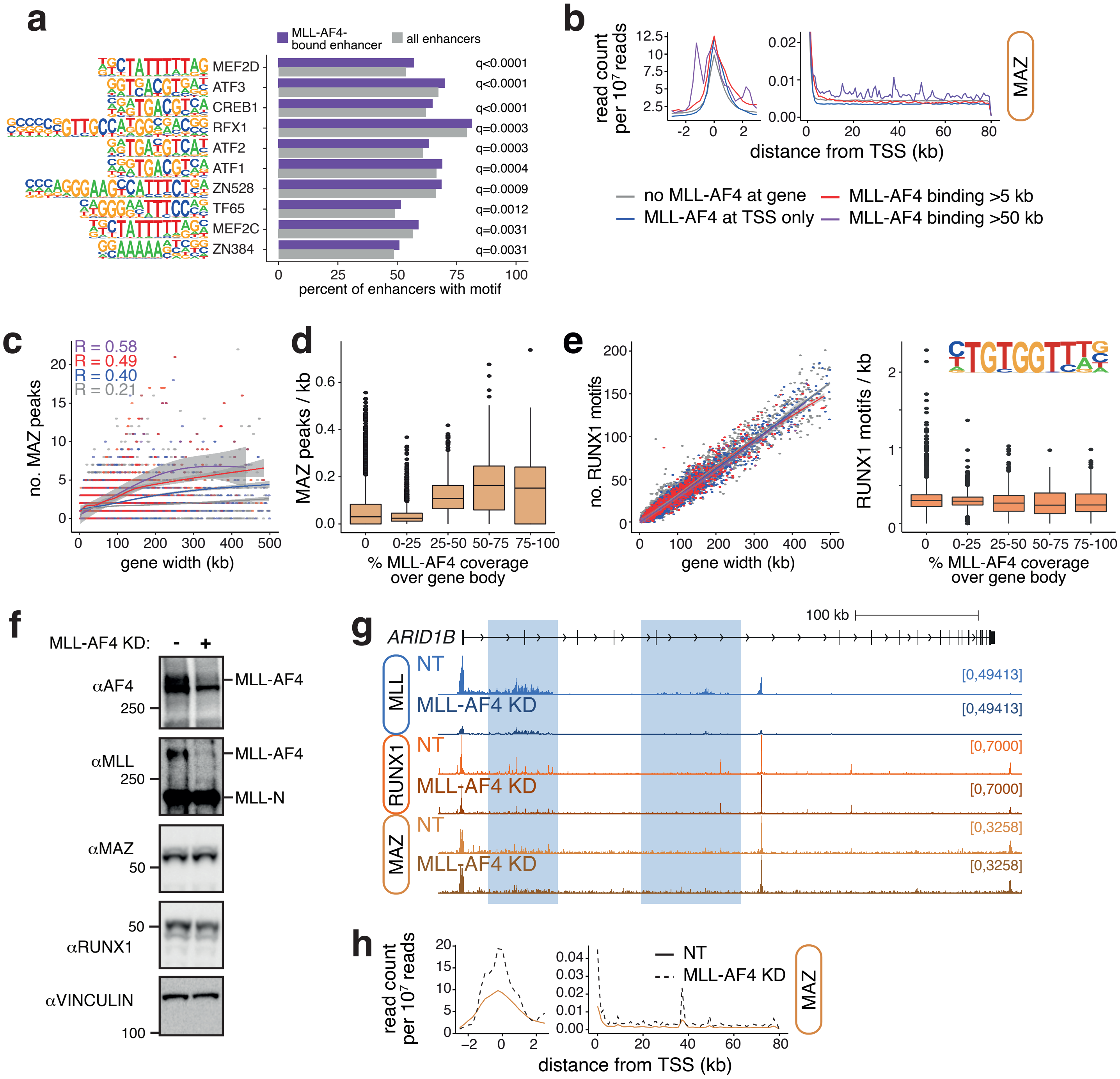

Supplementary Figure 9.

a, Enrichment of transcription factor motifs at all enhancers and the unified MLL-AF4-bound enhancer set in MLL-AF4 patients. **b**, Mean MAZ ChIP-seq signal at expressed promoters over a 6 kb (*left*) or 80 kb (*right*) window. Profiles stratified by MLL-AF4 binding status. Gene body ChIP-seq traces (*right*) are scaled to emphasize non-TSS binding. **c**, Relationship between MAZ peak frequency within gene body and gene body length, stratified by MLL-AF4 binding status as in (b). Local regression (LOESS) lines fit with 95% confidence interval in grey. Correlation (R) calculated by MLL-AF4 binding status. **d**, Density of MAZ ChIP-seq peaks over gene bodies, stratified by proportion of MLL-AF4 coverage, n=1. Midline shows median, with upper and lower hinges showing 25th and 75th percentile, respectively. Upper and lower hinges extend to the largest and smallest datapoints within 1.5 times the interquartile range of either hinge. **e**, *Left*, relationship between RUNX1 motif frequency within gene body and gene body length, stratified by MLL-AF4 binding status. Local regression (LOESS) lines fit with 95% CI in gray. *Right*, density of RUNX1 motifs over gene bodies, stratified by proportion of MLL-AF4 coverage. HOMER RUNX1 motif logo shown. **f**, Western blot for AF4 (detecting MLL-AF4), wild-type MLL, MAZ and RUNX1 in control (non-targeting; -) and 48h MLL-AF4 knockdown (+) SEM cells. VINCULIN is shown as a loading control. Blots are representative of two replicates. Source data are provided at the end of this document. **g**, ChIP-seq for MLL, RUNX1 and MAZ at *ARID1B* under control (NT) and 48h MLL-AF4 knockdown conditions. Putative enhancers are highlighted. **h**, Mean MAZ ChIP-seq signal under control (NT) and 48h MLL-AF4 knockdown conditions, at expressed promoters of genes containing an MLL-AF4 binding domain >50 kb, over a 6 kb (*left*) or 80 kb (*right*) window.

Supplementary Tables

Supplementary Table 1. High-throughput data collected from MLL-AF4 ALL patient samples.

Supplementary Table 2. Gene promoters analyzed by NG Capture-C and MCC.

Supplementary Table 3. Antibodies used in this study.

Supplementary Table 4. Primers and oligonucleotides used in this study.

Supplementary Table 5. Datasets used in this study.

Supplementary Table 1. High-throughput data collected from MLL-AF4 ALL patient samples.

Sample	Dataset		Source
**chALL #1**	ChIP-seq	H3K4me1	GSE135024
		H3K4me3	
		H3K27ac	
		H3K79me2	
		MLL	This study
		AF4	
	TOPmentation	MLL	This study
	ATAC-seq		GSE135024
	polyA RNA-seq		This study
**chALL #2**	ChIP-seq	MLL	GSE151390
		AF4	
	TOPmentation	H3K4me1	This study
		H3K4me3	
		H3K27ac	
		H3K79me2	
	ATAC-seq		This study
	polyA RNA-seq		This study
**chALL #3**	ChIP-seq	MLL	This study
		AF4	
	TOPmentation	MLL	This study
		PAF1	
		RUNX1	
		H3K4me1	
		H3K4me3	
		H3K27ac	
		H3K79me2	
		H3K27me3	
	ATAC-seq		This study
	polyA RNA-seq		This study
**iALL #1**	ChIP-seq	H3K79me3	GSE135024
		MLL	GSE83671
		AF4	
**iALL #2**	ChIP-seq	MLL	This study
	TOPmentation	MLL	This study
		RUNX1	This study
		H3K4me1	
		H3K4me3	
		H3K27ac	
		H3K79me2	
		H3K27me3	
	ATAC-seq		This study
	polyA RNA-seq		This study

Supplementary Table 2. Gene promoters analyzed by NG Capture-C and MCC.

Gene	Experiment	Probe coordinates (hg19)		
		Chromosome	Start	End
ARID1B	NG Capture-C	6	157099820	157100601
ASH2L		8	37961940	37963209
BAZ2B		2	160472165	160472912
BCL11A		2	60778931	60782448
BCL2		18	60986641	60988918
CDK6		7	92462911	92463843
ELL2		5	95297309	95297978
EP300		22	41487595	41490012
EZH2		7	148580112	148580317
FLT3		13	28674038	28675050
FOS		14	75744773	75746081
FUT10		8	33330317	33330678
GADD45A		1	68150066	68151771
JARID1A		12	498164	499589
JMJD1C		10	65226055	65226262
JUN		1	59249099	59250691
LMO4		1	87793884	87795360
MBNL1		3	151986212	151986427
		3	151986424	151989271
MED13		17	60141972	60142862
		17	60142859	60143711
MEF2C		5	88180024	88180319
MSL3		X	11774388	11776889
MYB		6	135501733	135502770
MYC		8	128748253	128748439
OGT		X	70752518	70753336
PROM1		4	16084464	16085645
		4	16077645	16078021
SMYD2		1	214454064	214456235
SPI1		11	47399928	47400398
SUPT3H		6	45345424	45345962
SUZ12		17	29058515	29059304
TAPT1		4	16226868	16228644
TET2		4	106067209	106069662
		4	106066532	106067212
ARID1B	MCC	6	157099022	157099142
CDK6		7	92463218	92463338
FLT3		13	28674652	28674772
IKZF1		7	50344187	50344307
LMO4		1	87794067	87794187
MYC		8	128748383	128748503
SMAD3		15	67358205	67358325

Supplementary Table 3. Antibodies used in this study.

Target	Catalogue Number	Company	WB dilution
H3K4me1	pAb-194-050	Diagenode	
H3K4me3	39159	Active Motif	
H3K27ac	C15410196	Diagenode	
H3K79me2	04-835	Millipore	
H3K79me3	C15410068	Diagenode	
H3K27me3	07-449	Millipore	
MLL	A300-086A	Bethyl	1/5000
AF4	ab31812	Abcam	1/1000
ENL	A302-268A	Bethyl	
PAF1	A300-172A/1	Bethyl	1/5000
SSRP1	A303-068A	Bethyl	1/10000
SPT16	sc-28734	Santa Cruz	1/500
HCFC1	A301-400A	Bethyl	1/2000
CTR9	A301-395A	Bethyl	1/5000
RUNX1 (WB)	4334	Cell Signaling Technology	1/1000
RUNX1 (ChIP)	ab23980	Abcam	
MAZ	A301-652A	Bethyl	1/1000
GAPDH	A300-641A	Bethyl	1/10000
VINCULIN	4650	Cell Signaling Technology	1/10000
Anti-FLAG-conjugated beads	M8823	Sigma	
FS2 (TetR)	Gift of Prof Rob Klose (University of Oxford)		

Supplementary Table 4. Primers and oligonucleotides used in this study.

Experiment	Name	Sequence
ChIP-qPCR primers	*ARID1B* SEM enhancer F	TTTCCAGTTCCGTCCAAGTATT
	ARID1B SEM enhancer R	CTTTCTCCCAGCTTCCTGTTAG
	ARID1B RS4;11 enhancer F	CATGTCATACGCTGTGTCAGA
	ARID1B RS4;11 enhancer R	CCCACGTGTCCATAGAAAGATAA
	GNAQ SEM enhancer F	GAGGTGGAAGTCAAAGCAAATG
	GNAQ SEM enhancer R	AGCAGGTGTTGGTGTTCTT
	GNAQ common enhancer F	AGTCAACAACAGACCACGTAAA
	GNAQ common enhancer R	TACGATGTCAGTAGGCGATAGG
	MEF2C SEM enhancer F	TTCCACACCCTGTTGCTATG
	MEF2C SEM enhancer R	TGTGTGTGTGTATGCCAGTT
	Negative F	GGCTCCTGTAACCAACCACTACC
	Negative R	CCTCTGGGCTGGCTTCATTC
qRT-PCR Taqman probes	*ARID1B*	Hs00368175_m1
	CDK6	Hs01026371_m1
	GNAQ	Hs01586104_m1
	MEF2C	Hs00231149_m1
	YWHAZ	Hs03044281_g1
CRISPR sgRNAs	*PAF1* C-term F	caccAGTGACAGTGACTGAGTCCC
	PAF1 C-term R	aaacGGGACTCAGTCACTGTCACT
	SSRP1 C-term F	caccGATCCGATGAGTAGAAACGG
	SSRP1 C-term R	aaacCCGTTTCTACTCATCGGATC

Supplementary Table 5. Datasets used in this study.

Cell line	Details	Source*
**RNA-seq**		
*MLL*r and *MLL*wt iBCP-ALL patient samples		European Nucleotide Archive, PRJEB23605
**Microarray expression data**		
ECOG E2993		GSE34861
COG P9906		GSE11877
St. Jude 2003		http://www.stjuderesearch.org/data/ALL3
St. Jude 2013		GSE26281
**Nascent RNA-seq**		
SEM	MLL-AF4 NT/KD	GSE85988
**Transient transcriptome-seq**		
SEM	PAF1-FKBP ±dTAG-13	This study
	SSRP1-FKBP ±dTAG-13	This study
MM1.S	PAF1-FKBP ±dTAG-13	This study
**ChIP-seq**		
SEM	H3K4me1	GSE74812
	H3K4me3	GSE74812
	H3K27ac	GSE74812
	H3K79me3	GSE74812
	MLL	GSE74812
	AF4	GSE74812
	MED1	GSE83671
	SSRP1	This study
	ENL	GSE74812
	MENIN	GSE83671
	PAF1	GSE83671
	LEO1	GSE83671
	PAF1 MLL-AF4 NT/KD	This study
	H3K79me3 MLL-AF4 NT/KD	This study
	H3K27ac MLL-AF4 NT/KD	This study
	RUNX1 MLL-AF4 NT/KD	This study
	MAZ MLL-AF4 NT/KD	This study
	H3K27ac PAF1-FKBP ±dTAG-13	This study
	MLL PAF1-FKBP ±dTAG-13	This study
	H3K27ac SSRP1-FKBP ±dTAG-13	This study
	MLL SSRP1-FKBP ±dTAG-13	This study
RS4;11	H3K4me1	GSE71616
	H3K4me3	GSE71616
	H3K27ac	GSE71616
	MLL	GSE151390
	AF4	GSE151390
MM1.S	PAF1	This study
	H3K27ac PAF1-FKBP ±dTAG-13	This study
	H3K4me1	GSE95917
	BRD4	GSE44931
	MED1	GSE44931

KOPN8	MLL	GSE83671
ML2	MLL	GSE95511
MV4;11	MLL	GSE83671
	H3K27ac	GSE79899
THP1	MLL	GSE83671
	H3K27ac	GSE117865
SHI1	MLL	GSE95511
CD34+ cord blood	FLAG-MLL-Af4	GSE84116
ALL cell lines	H3K27ac	GSE186941
**TOPmentation**		
SEM	PAF1 PAF1-FKBP ±dTAG-13	This study
	H3K79me3 PAF1-FKBP ±dTAG-13	This study
	H3K79me3 SSRP1-FKBP ±dTAG-13	This study
RS4;11	ENL	This study
	PAF1	This study
**HT-ChIPmentation**		
RS4;11	ENL	This study
	PAF1	This study
**ATAC-seq**		
SEM		GSE117865
RS4;11		GSE117865
Sorted adult human blood cell populations		GSE74912
**NG Capture-C**		
SEM	DMSO/DOT1Li	GSE117865
SEM	DMSO/AT1 (BRD4 PROTAC)	GSE139437
SEM	MLL-AF4 NT/KD	This study
SEM	PAF1 degron ±dTAG-13	This study
SEM	SSRP1 degron ±dTAG-13	This study
RS4;11		GSE117865
**Micro-Capture-C**		
SEM		This study

* GEO accession numbers unless otherwise indicated

Supplementary References

1. Crump, N.T. *et al.* BET inhibition disrupts transcription but retains enhancer-promoter contact. *Nat Commun* **12**, 223 (2021).

2. Kodgule, R. *et al.* ETV6 Deficiency Unlocks ERG-Dependent Microsatellite Enhancers to Drive Aberrant Gene Activation in B-Lymphoblastic Leukemia. *Blood Cancer Discov* **4**, 34-53 (2023).

3. Corces, M.R. *et al.* Lineage-specific and single-cell chromatin accessibility charts human hematopoiesis and leukemia evolution. *Nat Genet* **48**, 1193-203 (2016).

4. Agraz-Doblas, A. *et al.* Unraveling the cellular origin and clinical prognostic markers of infant B-cell acute lymphoblastic leukemia using genome-wide analysis. *Haematologica* **104**, 1176-1188 (2019).

5. Geng, H. *et al.* Integrative Epigenomic Analysis Identifies Biomarkers and Therapeutic Targets in Adult B-Acute Lymphoblastic Leukemia. *Cancer Discov* **2**, 1004-1023 (2012).

6. Harvey, R.C. *et al.* Identification of novel cluster groups in pediatric high-risk B-precursor acute lymphoblastic leukemia with gene expression profiling: correlation with genome-wide DNA copy number alterations, clinical characteristics, and outcome. *Blood* **116**, 4874-84 (2010).

7. Ross, M.E. *et al.* Classification of pediatric acute lymphoblastic leukemia by gene expression profiling. *Blood* **102**, 2951-9 (2003).

8. Figueroa, M.E. *et al.* Integrated genetic and epigenetic analysis of childhood acute lymphoblastic leukemia. *J Clin Invest* **123**, 3099-111 (2013).

9. Godfrey, L. *et al.* DOT1L inhibition reveals a distinct subset of enhancers dependent on H3K79 methylation. *Nat Commun* **10**, 2803 (2019).
