# Supplementary material for: MLL-AF4 cooperates with PAF1 and FACT to drive high-density enhancer interactions in leukemia
Source: Nat Commun. 2023 Aug 25;14:5208. doi: 10.1038/s41467-023-40981-9 (PMC10457349; doi:10.1038/s41467-023-40981-9)
Supplement: Supplementary file 3 — Description of Additional Supplementary Files [file 41467_2023_40981_MOESM3_ESM.pdf]

Description of Additional Supplementary Files

File Name: Supplementary Data 1
Description: MLL-AF4 bound enhancers common between MLL-AF4 ALL patient samples, annotated with nearest gene.

File Name: Supplementary Data 2
Description: Proteins identified in IP-MS screen.
