# Supplementary material for: MLL-AF4 cooperates with PAF1 and FACT to drive high-density enhancer interactions in leukemia
Source: Nat Commun. 2023 Aug 25;14:5208. doi: 10.1038/s41467-023-40981-9 (PMC10457349; doi:10.1038/s41467-023-40981-9)
Supplement: Supplementary file 6 — Reporting Summary [file 41467_2023_40981_MOESM6_ESM.pdf]

nature portfolio

Corresponding author(s): Thomas A Milne and Nicholas T Crump

Last updated by author(s): Aug 17, 2023

Reporting Summary

Nature Portfolio wishes to improve the reproducibility of the work that we publish. This form provides structure for consistency and transparency in reporting. For further information on Nature Portfolio policies, see our Editorial Policies and the Editorial Policy Checklist.

Statistics

For all statistical analyses, confirm that the following items are present in the figure legend, table legend, main text, or Methods section.

n/a	Confirmed	
☐	☒	The exact sample size (*n*) for each experimental group/condition, given as a discrete number and unit of measurement
☐	☒	A statement on whether measurements were taken from distinct samples or whether the same sample was measured repeatedly
☐	☒	The statistical test(s) used AND whether they are one- or two-sided<br>*Only common tests should be described solely by name; describe more complex techniques in the Methods section.*
☐	☒	A description of all covariates tested
☐	☒	A description of any assumptions or corrections, such as tests of normality and adjustment for multiple comparisons
☐	☒	A full description of the statistical parameters including central tendency (e.g. means) or other basic estimates (e.g. regression coefficient) AND variation (e.g. standard deviation) or associated estimates of uncertainty (e.g. confidence intervals)
☐	☒	For null hypothesis testing, the test statistic (e.g. *F*, *t*, *r*) with confidence intervals, effect sizes, degrees of freedom and *P* value noted<br>*Give P values as exact values whenever suitable.*
☒	☐	For Bayesian analysis, information on the choice of priors and Markov chain Monte Carlo settings
☒	☐	For hierarchical and complex designs, identification of the appropriate level for tests and full reporting of outcomes
☐	☒	Estimates of effect sizes (e.g. Cohen's *d*, Pearson's *r*), indicating how they were calculated

Our web collection on statistics for biologists contains articles on many of the points above.

Software and code

Policy information about availability of computer code

Data collection

Next-generation sequencing (Illumina). No software was used for data collection.

Data analysis

For ChIP-seq and ATAC-seq: Following QC of FASTQ files by FastQC (v0.12.1; http://www.bioinformatics.babraham.ac.uk/projects/fastqc/), reads were trimmed using trim_galore with Cutadapt (v0.6.10; https://www.bioinformatics.babraham.ac.uk/projects/trim_galore/). Trimmed reads were then mapped to the hg19 reference genome using Bowtie 2 (v2.5.1). PCR duplicates were removed using picard MarkDuplicates (v3.0.0; http://broadinstitute.github.io/picard). Problematic genomic regions present in the ENCODE Blacklist (https://doi.org/10.1038/s41598-019-45839-z) were removed from the aligned files and further QC of the aligned files was performed using samtools (v1.17). As many of the factors that were immunoprecipitated have a mix of both sharp and broad modalities, we used either HOMER (v4.11) or the deep learning based peak caller LanceOtron (v1.0.8; with a peak score cut-off value of 0.5) to call peaks. Due to the limited material available, replicates were not performed and therefore an IDR based method was not used. BigWigs were generated using the deepTools (v3.5.1) bamCoverage command, with the flags –extendReads –normalizeUsing RPKM, and visualized in the UCSC genome browser.

For RNA-seq: Reads were subjected to quality checking by fastQC (v0.12.1) and trimming using trim_galore (v0.6.10) to remove contaminating sequencing adapters, poor quality reads and reads shorter than 21 bp. Reads were then aligned to hg19 using STAR (v2.4.2a) in paired end mode using default parameters. Gene expression levels were quantified as read counts using the featureCounts function from the Subread package (v2.0.2) with default parameters. The read counts were used to identify differential gene expression between conditions using the DESeq2 (v3.12) package.

Microarray data was normalized with RMA method using Expression ConsoleTM software (Version 1.1, Affymetrix, Santa Clara, CA) for the Affymetrix arrays HG-U133 plus2 (COG data) or NimbleScan software (version 2.5, Roche NimbleGen, Madison, WI) for the NimbleGen arrays HG18 60mer expression 385K platform (ECOG data). Downstream microarray analysis was performed using R version 2.14.0. Heatmaps were

generated with Cluster/TreeView3.0 (http://bonsai.hgc.jp/~mdehoon/software/cluster/software.htm).

Capture-C analysis was performed using CapCruncher v0.2.3 (https://doi.org/10.5281/zenodo.6326102).

Micro-Capture-C analysis was performed using the MCC pipeline (https://github.com/jojdavies/Micro-Capture-C).

For manuscripts utilizing custom algorithms or software that are central to the research but not yet described in published literature, software must be made available to editors and reviewers. We strongly encourage code deposition in a community repository (e.g. GitHub). See the Nature Portfolio guidelines for submitting code & software for further information.

Data

Policy information about availability of data

All manuscripts must include a data availability statement. This statement should provide the following information, where applicable:

- Accession codes, unique identifiers, or web links for publicly available datasets
- A description of any restrictions on data availability
- For clinical datasets or third party data, please ensure that the statement adheres to our policy

The raw high throughput sequencing data generated in this study have been deposited in the Gene Expression Omnibus (GEO) under accession number GSE202451 (SEM/AL; polyA-RNA-seq, ChIP-seq, ATAC-seq, TOPmentation seq, TT-seq and Capture-C data) and GSE236664 (Multiple Myeloma; ChIP-seq and TT-seq data). The raw mass spectrometry data generated in this study were deposited to the ProteomeXchange Consortium via the PRIDE partner repository with the dataset identifier PXD043920.
The publicly available cell line ChIP-seq data reused in this study are available in GEO under accession codes GSE74812, GSE8367, GSE71616, GSE151390, GSE959171, GSE44931, GSE95511, GSE79899, GSE117865, GSE84116 and GSE18694. The publicly available ATAC-seq data reused in this study are available in GEO under accession codes GSE117865 and GSE74912. The publicly available SEM nascent RNA-seq data reused in this study are available in GEO under accession code GSE85988. The publicly available Next Generation Capture-C data reused in this study are available in GEO under accession codes GSE117865 and GSE139437. The publicly available ALL patient ChIP-seq data reused in this study are available in GEO under accession codes GSE13502441, GSE15139083 and GSE83671. The publicly available ALL patient RNA-seq data reused in this study are available from the European Nucleotide Archive under accession PRJEB23605 and microarray expression data are available in GEO under accession codes GSE34861, GSE11877, GSE26281 and at http://www.stjuderesearch.org/data/ALL3.
The remaining data are available within the Article, Supplementary Information or Source Data file. Source data are provided with this paper.

Human research participants

Policy information about studies involving human research participants and Sex and Gender in Research.

Reporting on sex and gender	The samples in this paper are three male and one female. Owing to the small sample size, analysis of sex-specific effects was not possible in this study.
Population characteristics	Acute leukemia patient samples were obtained from Blood Cancer UK Childhood Leukaemia Cell Bank (now VIVO Biobank), UK. All samples were anonymised at source, provided a unique study number and linked. The only population characteristics available to us were age and sex. Four patient samples from the UK CellBank were analyzed (three childhood, 1-18 years old and one infant, ≤ 1 year old).
Recruitment	Acute leukemia patient samples were obtained from Blood Cancer UK Childhood Leukaemia Cell Bank (now VIVO Biobank), UK after appropriate review of our research project to ensure that it was covered under their ethics approval (REC: 16/SW/0219). Cell Bank/VIVO Biobank focuses on samples of childhood cancer only, based on availability and informed consent provided by either the individuals or the appropriate guardian. Samples were requested by the Milne/Roy labs based on the presence of an MLL rearrangement and the availability of live cells, with no specific consideration given for age or sex. The data will be biased towards MLL rearrangements in a childhood/infant context and are not applicable to older patients. The small sample size has thus far prevented the study of sex or gender specific effects.
Ethics oversight	UK Human Tissue Authority (HTA, www.hta.gov.uk). Ethical approval: NHS HRA: REC: 16/SW/0219. All patients give informed consent via a specific CellBank consent form as found at https://cellbank.org.uk/participants. Informed consent was obtained from all participants or those with parental responsibility, and participants did not receive any monetary compensation. The consent is held by CellBank and the treating hospital.

Note that full information on the approval of the study protocol must also be provided in the manuscript.

Field-specific reporting

Please select the one below that is the best fit for your research. If you are not sure, read the appropriate sections before making your selection.

☒ Life sciences ☐ Behavioural & social sciences ☐ Ecological, evolutionary & environmental sciences

For a reference copy of the document with all sections, see nature.com/documents/nr-reporting-summary-flat.pdf

Life sciences study design

All studies must disclose on these points even when the disclosure is negative.

Sample size	Statistical methods were not used to assign sample size. Samples sizes were determined based on previous studies performed in the lab using similar techniques to enable reasonable statistical analysis (Kerry et al. 2017, Godfrey et al. 2019, Godfrey et al. 2021, Crump et al. 2021). Experiments were performed with 3-4 biological replicates as is common in the field (e.g. Rose et al 2016 PMID: 27705745; Blackledge et al 2020 PMID: 31883950; Hughes et al 2023 PMID: 36759609). The observed biological effects of interest were consistent between replicates.
Data exclusions	No data were excluded from this analysis
Replication	ChIP-seq and TOPmentation data represent a single biological replicate, with peaks and troughs of signal at specific loci confirmed by ChIP-qPCR. ChIP-qPCR experiments were conducted with multiple biological replicates to confirm any changes following treatment.<br>Capture-C experiments were conducted in triplicate, with averaged data presented. Statistical difference between treatments were assessed by Holm-Bonferroni adjusted p-values from paired Mann-Whitney test.<br>TT-seq experiments in SEM cells were conducted in triplicate, with averaged data presented. Statistical difference between treatments were assessed using EdgeR. A single TT-seq replicate was conducted in MM1.S cells.<br>The numbers of biological replicates for each experiment (typically 3-4) are given in the Methods section and/or in the figure legends.<br>All attempts at replication were successful.
Randomization	Randomization was not used in this study as it includes only molecular assays performed in cells of known genotype.
Blinding	Investigators were not blinded as this was not relevant to analysis of the data generated here and there were no prior assumptions about experimental outcomes. The same pipelines and scripts were used to analyze all samples uniformly.

Reporting for specific materials, systems and methods

We require information from authors about some types of materials, experimental systems and methods used in many studies. Here, indicate whether each material, system or method listed is relevant to your study. If you are not sure if a list item applies to your research, read the appropriate section before selecting a response.

Materials & experimental systems

n/a	Involved in the study	
☐	☒	Antibodies
☐	☒	Eukaryotic cell lines
☒	☐	Palaeontology and archaeology
☒	☐	Animals and other organisms
☒	☐	Clinical data
☒	☐	Dual use research of concern

Methods

n/a	Involved in the study	
☐	☒	ChIP-seq
☒	☐	Flow cytometry
☒	☐	MRI-based neuroimaging

Antibodies

Antibodies used	anti-AF4: Abcam ab31812 lot GR33890-1; ChIP (1/500), WB (1/1000)<br>anti-ENL: Bethyl A302-268A lot 1; ChIP (1/500)<br>anti-H3K27ac: Diagenode C15410196 lot A1723-0041D; ChIP (1/1000)<br>anti-H3K27me3: Millipore 07-449 lot 2318778; ChIP (1/500)<br>anti-H3K4me1: Diagenode C15410194 lot A1862D; ChIP (1/500)<br>anti-H3K4me3: Active Motif 39159; ChIP (1/500)<br>anti-H3K79me2: Millipore 04-835 lot NG1800794; ChIP (1/500)<br>anti-H3K79me3: Diagenode C15410068; ChIP (1/500)<br>anti-MLL: Bethyl A300-086A lot 6; ChIP (1/500), WB (1/5000)<br>anti-PAF1: Bethyl A300-172A lot 3; ChIP (1/500), WB (1/5000)<br>anti-SSRP1: Bethyl A303-067A lot 1; ChIP (1/500), WB (1/10000)<br>anti-SPT16: Santa Cruz sc-28734; WB (1/500)<br>anti-HCFC1: Bethyl A301-400A; WB (1/2000)<br>anti-CTR9: Bethyl A301-395A; WB (1/5000)<br>anti-RUNX1 (WB): Cell Signaling 4334; WB (1/1000)<br>anti-RUNX1 (ChIP-seq): Abcam ab23980; ChIP (1/500)<br>anti-MAZ: Bethyl A301-652A; ChIP (1/500), WB (1/1000)<br>anti-GAPDH: Bethyl A300-641A; WB (1/10000)<br>anti-VINCULIN: Cell Signaling 4650; WB (1/10000)<br>anti-FLAG beads: Sigma-Aldrich M8823 clone M2
Validation	anti-AF4 (ChIP-seq, TOPmentation, ChIP-qPCR and Western blotting): Validated for ChIP in-house by ChIP-qPCR, validated for WB in human cell lines on the manufacturer's website

anti-ENL (TOPmentation and ChIP-qPCR): Validated for ChIP in-house by ChIP-qPCR
anti-H3K27ac (ChIP-seq, TOPmentation and ChIP-qPCR): Validated for ChIP in human cell lines on the manufacturer's website
anti-H3K27me3 (TOPmentation): Validated for ChIP in human cell lines on the manufacturer's website
anti-H3K4me1 (TOPmentation): Validated for ChIP in human cell lines on the manufacturer's website
anti-H3K4me3 (TOPmentation): Validated for ChIP in human cell lines on the manufacturer's website
anti-H3K79me2 (TOPmentation): Validated for ChIP in human cell lines on the manufacturer's website
anti-H3K79me3 (ChIP-seq and TOPmentation): Validated for ChIP in-house by ChIP-qPCR
anti-MLL (ChIP-seq, TOPmentation, ChIP-qPCR and Western blotting): Validated for ChIP in-house by ChIP-qPCR, validated for WB in human cell lines on the manufacturer's website
anti-PAF1 (ChIP-seq, TOPmentation and ChIP-qPCR): Validated for ChIP in-house by ChIP-qPCR
anti-SSRP1 (ChIP-seq and ChIP-qPCR): Validated for ChIP in-house by ChIP-qPCR
anti-SPT16 (Western blotting): Validated for WB in human cell lines on the manufacturer's website
anti-HCFC1 (Western blotting): Validated for WB in human cell lines on the manufacturer's website
anti-CTR9 (Western blotting): Validated for WB in human cell lines on the manufacturer's website
anti-RUNX1 (Western blotting): Validated for WB in human cell lines on the manufacturer's website
anti-RUNX1 (ChIP-seq): Validated for ChIP in-house by ChIP-qPCR
anti-MAZ (Western blotting and ChIP-seq): Validated for WB in human cell lines on the manufacturer's website, validated for ChIP in-house by ChIP-qPCR
anti-GAPDH (Western blotting): Validated for WB in human cell lines on the manufacturer's website
anti-VINCULIN (Western blotting): Validated for WB in human cell lines on the manufacturer's website
anti-FLAG beads (immunoprecipitation): Validated for IP on the manufacturer's website

Eukaryotic cell lines

Policy information about cell lines and Sex and Gender in Research

Cell line source(s)

SEM cells (female; ACC 546) were purchased from DSMZ (www.cell-lines.de). RS4;11 (female; CRL-1873) and MM1.S (female; CRL-2974) cells were purchased from ATCC (www.lgcstandards-atcc.org). Mouse ES cells with a TetO-array (TOT2N mESC; male) were kindly provided by Prof. Rob Klose (University of Oxford; Blackledge et al 2014 PMID 24856970). Details on other cell lines associated with previously published data used in this study are available from the original publications associated with those datasets.

Authentication

Cells were validated by the supplier by STR DNA typing

Mycoplasma contamination

All cell lines were confirmed to be mycoplasma free

Commonly misidentified lines
(See ICLAC register)

None

ChIP-seq

Data deposition

☒ Confirm that both raw and final processed data have been deposited in a public database such as GEO.

☒ Confirm that you have deposited or provided access to graph files (e.g. BED files) for the called peaks.

Data access links
May remain private before publication.

https://www.ncbi.nlm.nih.gov/geo/query/acc.cgi?acc=GSE202451
https://www.ncbi.nlm.nih.gov/geo/query/acc.cgi?acc=GSE236664

Files in database submission

chALL1_Input_ChIP_R1.fastq.gz
chALL1_MLL_ChIP_R1.fastq.gz
chALL1_AF4_ChIP_R1.fastq.gz
chALL3_Input_ChIP_R1.fastq.gz
chALL3_MLL_ChIP_R1.fastq.gz
chALL3_AF4_ChIP_R1.fastq.gz
iALL2_Input_ChIP_R1.fastq.gz
iALL2_MLL_ChIP_R1.fastq.gz
chALL1_Input_ChIP_R2.fastq.gz
chALL1_MLL_ChIP_R2.fastq.gz
chALL1_AF4_ChIP_R2.fastq.gz
chALL3_Input_ChIP_R2.fastq.gz
chALL3_MLL_ChIP_R2.fastq.gz
chALL3_AF4_ChIP_R2.fastq.gz
iALL2_Input_ChIP_R2.fastq.gz
iALL2_MLL_ChIP_R2.fastq.gz
chALL1_Input_ChIP.bigWig
chALL1_MLL_ChIP.bigWig
chALL1_AF4_ChIP.bigWig
chALL3_Input_ChIP.bigWig
chALL3_MLL_ChIP.bigWig
chALL3_AF4_ChIP.bigWig
iALL2_Input_ChIP.bigWig
iALL2_MLL_ChIP.bigWig

chALL1_RNA_R1.fastq.gz
chALL1_RNA_R2.fastq.gz
chALL2_RNA_R1.fastq.gz
chALL2_RNA_R2.fastq.gz
chALL3_RNA_R1.fastq.gz
chALL3_RNA_R2.fastq.gz
iALL2_RNA_R1.fastq.gz
iALL2_RNA_R2.fastq.gz
ALL_patient_RNAseq_cpm.txt.gz
chALL2_ATAC_R1.fastq.gz
chALL2_ATAC_R2.fastq.gz
chALL3_ATAC_R1.fastq.gz
chALL3_ATAC_R2.fastq.gz
iALL2_ATAC_R1.fastq.gz
iALL2_ATAC_R2.fastq.gz
chALL2_ATAC.bigWig
chALL3_ATAC.bigWig
iALL2_ATAC.bigWig
chALL1_MLL_TOPM_R1.fastq.gz
chALL1_MLL_TOPM_R2.fastq.gz
chALL2_H3K27ac_TOPM_R1.fastq.gz
chALL2_H3K27ac_TOPM_R2.fastq.gz
chALL2_H3K4me1_TOPM_R1.fastq.gz
chALL2_H3K4me1_TOPM_R2.fastq.gz
chALL2_H3K4me3_TOPM_R1.fastq.gz
chALL2_H3K4me3_TOPM_R2.fastq.gz
chALL2_H3K79me2_TOPM_R1.fastq.gz
chALL2_H3K79me2_TOPM_R2.fastq.gz
chALL3_H3K27ac_TOPM_R1.fastq.gz
chALL3_H3K27ac_TOPM_R2.fastq.gz
chALL3_H3K27me3_TOPM_R1.fastq.gz
chALL3_H3K27me3_TOPM_R2.fastq.gz
chALL3_H3K4me1_TOPM_R1.fastq.gz
chALL3_H3K4me1_TOPM_R2.fastq.gz
chALL3_H3K4me3_TOPM_R1.fastq.gz
chALL3_H3K4me3_TOPM_R2.fastq.gz
chALL3_H3K79me2_TOPM_R1.fastq.gz
chALL3_H3K79me2_TOPM_R2.fastq.gz
chALL3_MLL_TOPM_R1.fastq.gz
chALL3_MLL_TOPM_R2.fastq.gz
chALL3_PAF1_TOPM_R1.fastq.gz
chALL3_PAF1_TOPM_R2.fastq.gz
chALL3_RUNX1_TOPM_R1.fastq.gz
chALL3_RUNX1_TOPM_R2.fastq.gz
iALL2_H3K27ac_TOPM_R1.fastq.gz
iALL2_H3K27ac_TOPM_R2.fastq.gz
iALL2_H3K27me3_TOPM_R1.fastq.gz
iALL2_H3K27me3_TOPM_R2.fastq.gz
iALL2_H3K4me1_TOPM_R1.fastq.gz
iALL2_H3K4me1_TOPM_R2.fastq.gz
iALL2_H3K4me3_TOPM_R1.fastq.gz
iALL2_H3K4me3_TOPM_R2.fastq.gz
iALL2_H3K79me2_TOPM_R1.fastq.gz
iALL2_H3K79me2_TOPM_R2.fastq.gz
iALL2_MLL_TOPM_R1.fastq.gz
iALL2_MLL_TOPM_R2.fastq.gz
iALL2_RUNX1_TOPM_R1.fastq.gz
iALL2_RUNX1_TOPM_R2.fastq.gz
chALL1_MLL_TOPM.bigWig
chALL2_H3K27ac_TOPM.bigWig
chALL2_H3K4me1_TOPM.bigWig
chALL2_H3K4me3_TOPM.bigWig
chALL2_H3K79me2_TOPM.bigWig
chALL3_H3K27ac_TOPM.bigWig
chALL3_H3K27me3_TOPM.bigWig
chALL3_H3K4me1_TOPM.bigWig
chALL3_H3K4me3_TOPM.bigWig
chALL3_H3K79me2_TOPM.bigWig
chALL3_MLL_TOPM.bigWig
chALL3_PAF1_TOPM.bigWig
chALL3_RUNX1_TOPM.bigWig
iALL2_H3K27ac_TOPM.bigWig
iALL2_H3K27me3_TOPM.bigWig
iALL2_H3K4me1_TOPM.bigWig
iALL2_H3K4me3_TOPM.bigWig
iALL2_H3K79me2_TOPM.bigWig

iALL2_MLL_TOPM.bigWig
iALL2_RUNX1_TOPM.bigWig
SEM_SSRP1-Input_ChIP_R1.fastq.gz
SEM_SSRP1-Input_ChIP_R2.fastq.gz
SEM_SSRP1_ChIP_R1.fastq.gz
SEM_SSRP1_ChIP_R2.fastq.gz
SEM_H3K27ac-Input_NT_ChIP_R1.fastq.gz
SEM_H3K27ac-Input_NT_ChIP_R2.fastq.gz
SEM_H3K27ac_NT_ChIP_R1.fastq.gz
SEM_H3K27ac_NT_ChIP_R2.fastq.gz
SEM_H3K27ac-Input_MLLAF4-KD_ChIP_R1.fastq.gz
SEM_H3K27ac-Input_MLLAF4-KD_ChIP_R2.fastq.gz
SEM_H3K27ac_MLLAF4-KD_ChIP_R1.fastq.gz
SEM_H3K27ac_MLLAF4-KD_ChIP_R2.fastq.gz
SEM_H3K79me3-Input_NT_ChIP_R1.fastq.gz
SEM_H3K79me3-Input_NT_ChIP_R2.fastq.gz
SEM_H3K79me3_NT_ChIP_R1.fastq.gz
SEM_H3K79me3_NT_ChIP_R2.fastq.gz
SEM_H3K79me3-Input_MLLAF4-KD_ChIP_R1.fastq.gz
SEM_H3K79me3-Input_MLLAF4-KD_ChIP_R2.fastq.gz
SEM_H3K79me3_MLLAF4-KD_ChIP_R1.fastq.gz
SEM_H3K79me3_MLLAF4-KD_ChIP_R2.fastq.gz
SEM_PAF1-Input_NT_ChIP_R1.fastq.gz
SEM_PAF1-Input_NT_ChIP_R2.fastq.gz
SEM_PAF1_NT_ChIP_R1.fastq.gz
SEM_PAF1_NT_ChIP_R2.fastq.gz
SEM_PAF1-Input_MLLAF4-KD_ChIP_R1.fastq.gz
SEM_PAF1-Input_MLLAF4-KD_ChIP_R2.fastq.gz
SEM_PAF1_MLLAF4-KD_ChIP_R1.fastq.gz
SEM_PAF1_MLLAF4-KD_ChIP_R2.fastq.gz
SEM-PAF1-FKBP_Histone-Input_DMSO_ChIP_R1.fastq.gz
SEM-PAF1-FKBP_Histone-Input_DMSO_ChIP_R2.fastq.gz
SEM-PAF1-FKBP_H3K27ac_DMSO_ChIP_R1.fastq.gz
SEM-PAF1-FKBP_H3K27ac_DMSO_ChIP_R2.fastq.gz
SEM-PAF1-FKBP_Histone-Input_dTag_ChIP_R1.fastq.gz
SEM-PAF1-FKBP_Histone-Input_dTag_ChIP_R2.fastq.gz
SEM-PAF1-FKBP_H3K27ac_dTag_ChIP_R1.fastq.gz
SEM-PAF1-FKBP_H3K27ac_dTag_ChIP_R2.fastq.gz
SEM-PAF1-FKBP_MLL-Input_DMSO_ChIP_R1.fastq.gz
SEM-PAF1-FKBP_MLL-Input_DMSO_ChIP_R2.fastq.gz
SEM-PAF1-FKBP_MLL_DMSO_ChIP_R1.fastq.gz
SEM-PAF1-FKBP_MLL_DMSO_ChIP_R2.fastq.gz
SEM-PAF1-FKBP_MLL-Input_dTag_ChIP_R1.fastq.gz
SEM-PAF1-FKBP_MLL-Input_dTag_ChIP_R2.fastq.gz
SEM-PAF1-FKBP_MLL_dTag_ChIP_R1.fastq.gz
SEM-PAF1-FKBP_MLL_dTag_ChIP_R2.fastq.gz
SEM-SSRP1-FKBP_Histone-Input_DMSO_ChIP_R1.fastq.gz
SEM-SSRP1-FKBP_Histone-Input_DMSO_ChIP_R2.fastq.gz
SEM-SSRP1-FKBP_H3K27ac_DMSO_ChIP_R1.fastq.gz
SEM-SSRP1-FKBP_H3K27ac_DMSO_ChIP_R2.fastq.gz
SEM-SSRP1-FKBP_Histone-Input_dTag_ChIP_R1.fastq.gz
SEM-SSRP1-FKBP_Histone-Input_dTag_ChIP_R2.fastq.gz
SEM-SSRP1-FKBP_H3K27ac_dTag_ChIP_R1.fastq.gz
SEM-SSRP1-FKBP_H3K27ac_dTag_ChIP_R2.fastq.gz
SEM-SSRP1-FKBP_MLL-Input_DMSO_ChIP_R1.fastq.gz
SEM-SSRP1-FKBP_MLL-Input_DMSO_ChIP_R2.fastq.gz
SEM-SSRP1-FKBP_MLL_DMSO_ChIP_R1.fastq.gz
SEM-SSRP1-FKBP_MLL_DMSO_ChIP_R2.fastq.gz
SEM-SSRP1-FKBP_MLL-Input_dTag_ChIP_R1.fastq.gz
SEM-SSRP1-FKBP_MLL-Input_dTag_ChIP_R2.fastq.gz
SEM-SSRP1-FKBP_MLL_dTag_ChIP_R1.fastq.gz
SEM-SSRP1-FKBP_MLL_dTag_ChIP_R2.fastq.gz
SEM_SSRP1.bigWig
SEM_SSRP1-Input.bigWig
SEM_H3K27ac_NT_ChIP.bigWig
SEM_H3K27ac-Input_NT_ChIP.bigWig
SEM_H3K27ac_MLLAF4-KD_ChIP.bigWig
SEM_H3K27ac-Input_MLLAF4-KD_ChIP.bigWig
SEM_H3K79me3_NT_ChIP.bigWig
SEM_H3K79me3-Input_NT_ChIP.bigWig
SEM_H3K79me3_MLLAF4-KD_ChIP.bigWig
SEM_H3K79me3-Input_MLLAF4-KD_ChIP.bigWig
SEM_PAF1_NT_ChIP.bigWig
SEM_PAF1-Input_NT_ChIP.bigWig
SEM_PAF1_MLLAF4-KD_ChIP.bigWig
SEM_PAF1-Input_MLLAF4-KD_ChIP.bigWig

SEM-PAF1-FKBP_H3K27ac_DMSO_ChIP.bigWig
SEM-PAF1-FKBP_H3K27ac-Input_DMSO_ChIP.bigWig
SEM-PAF1-FKBP_H3K27ac_dTag_ChIP.bigWig
SEM-PAF1-FKBP_H3K27ac-Input_dTag_ChIP.bigWig
SEM-PAF1-FKBP_MLL_DMSO_ChIP.bigWig
SEM-PAF1-FKBP_MLL-Input_DMSO_ChIP.bigWig
SEM-PAF1-FKBP_MLL_dTag_ChIP.bigWig
SEM-PAF1-FKBP_MLL-Input_dTag_ChIP.bigWig
SEM-SSRP1-FKBP_H3K27ac_DMSO_ChIP.bigWig
SEM-SSRP1-FKBP_H3K27ac-Input_DMSO_ChIP.bigWig
SEM-SSRP1-FKBP_H3K27ac_dTag_ChIP.bigWig
SEM-SSRP1-FKBP_H3K27ac-Input_dTag_ChIP.bigWig
SEM-SSRP1-FKBP_MLL_DMSO_ChIP.bigWig
SEM-SSRP1-FKBP_MLL-Input_DMSO_ChIP.bigWig
SEM-SSRP1-FKBP_MLL_dTag_ChIP.bigWig
SEM-SSRP1-FKBP_MLL-Input_dTag_ChIP.bigWig
SEM-PAF1-FKBP_H3K79me3_DMSO_TOPM_R1.fastq.gz
SEM-PAF1-FKBP_H3K79me3_DMSO_TOPM_R2.fastq.gz
SEM-PAF1-FKBP_H3K79me3_dTag_TOPM_R1.fastq.gz
SEM-PAF1-FKBP_H3K79me3_dTag_TOPM_R2.fastq.gz
SEM-PAF1-FKBP_PAF1_DMSO_TOPM_R1.fastq.gz
SEM-PAF1-FKBP_PAF1_DMSO_TOPM_R2.fastq.gz
SEM-PAF1-FKBP_PAF1_dTag_TOPM_R1.fastq.gz
SEM-PAF1-FKBP_PAF1_dTag_TOPM_R2.fastq.gz
SEM-PAF1-FKBP_PAF1-Input_DMSO_TOPM_R1.fastq.gz
SEM-PAF1-FKBP_PAF1-Input_DMSO_TOPM_R2.fastq.gz
SEM-PAF1-FKBP_PAF1-Input_dTag_TOPM_R1.fastq.gz
SEM-PAF1-FKBP_PAF1-Input_dTag_TOPM_R2.fastq.gz
SEM-SSRP1-FKBP_H3K79me3_DMSO_TOPM_R1.fastq.gz
SEM-SSRP1-FKBP_H3K79me3_DMSO_TOPM_R2.fastq.gz
SEM-SSRP1-FKBP_H3K79me3_dTag_TOPM_R1.fastq.gz
SEM-SSRP1-FKBP_H3K79me3_dTag_TOPM_R2.fastq.gz
RS411_ENL_HT-CM_R1.fastq.gz
RS411_ENL_HT-CM_R2.fastq.gz
RS411_ENL_TOPM_R1.fastq.gz
RS411_ENL_TOPM_R2.fastq.gz
RS411_PAF1_HT-CM_R1.fastq.gz
RS411_PAF1_HT-CM_R2.fastq.gz
RS411_PAF1_TOPM_R1.fastq.gz
RS411_PAF1_TOPM_R2.fastq.gz
SEM-PAF1-FKBP_H3K79me3_DMSO_TOPM.bigWig
SEM-PAF1-FKBP_H3K79me3_dTag_TOPM.bigWig
SEM-PAF1-FKBP_PAF1-Input_DMSO_TOPM.bigWig
SEM-PAF1-FKBP_PAF1_DMSO_TOPM.bigWig
SEM-PAF1-FKBP_PAF1-Input_dTag_TOPM.bigWig
SEM-PAF1-FKBP_PAF1_dTag_TOPM.bigWig
SEM-SSRP1-FKBP_H3K79me3_DMSO_TOPM.bigWig
SEM-SSRP1-FKBP_H3K79me3_dTag_TOPM.bigWig
RS411_ENL_HT-CM.bigWig
RS411_ENL_TOPM.bigWig
RS411_PAF1_HT-CM.bigWig
RS411_PAF1_TOPM.bigWig
SEM-PAF1-FKBP_DMSO-1_TTseq_R1.fastq.gz
SEM-PAF1-FKBP_DMSO-1_TTseq_R2.fastq.gz
SEM-PAF1-FKBP_DMSO-2_TTseq_R1.fastq.gz
SEM-PAF1-FKBP_DMSO-2_TTseq_R2.fastq.gz
SEM-PAF1-FKBP_DMSO-3_TTseq_R1.fastq.gz
SEM-PAF1-FKBP_DMSO-3_TTseq_R2.fastq.gz
SEM-PAF1-FKBP_dTag-1_TTseq_R1.fastq.gz
SEM-PAF1-FKBP_dTag-1_TTseq_R2.fastq.gz
SEM-PAF1-FKBP_dTag-2_TTseq_R1.fastq.gz
SEM-PAF1-FKBP_dTag-2_TTseq_R2.fastq.gz
SEM-PAF1-FKBP_dTag-3_TTseq_R1.fastq.gz
SEM-PAF1-FKBP_dTag-3_TTseq_R2.fastq.gz
SEM-SSRP1-FKBP_DMSO-1_TTseq_R1.fastq.gz
SEM-SSRP1-FKBP_DMSO-1_TTseq_R2.fastq.gz
SEM-SSRP1-FKBP_DMSO-2_TTseq_R1.fastq.gz
SEM-SSRP1-FKBP_DMSO-2_TTseq_R2.fastq.gz
SEM-SSRP1-FKBP_DMSO-3_TTseq_R1.fastq.gz
SEM-SSRP1-FKBP_DMSO-3_TTseq_R2.fastq.gz
SEM-SSRP1-FKBP_dTag-1_TTseq_R1.fastq.gz
SEM-SSRP1-FKBP_dTag-1_TTseq_R2.fastq.gz
SEM-SSRP1-FKBP_dTag-2_TTseq_R1.fastq.gz
SEM-SSRP1-FKBP_dTag-2_TTseq_R2.fastq.gz
SEM-SSRP1-FKBP_dTag-3_TTseq_R1.fastq.gz
SEM-SSRP1-FKBP_dTag-3_TTseq_R2.fastq.gz

SEM-PAF1-FKBP_TTseq_featureCounts.txt.gz
SEM-PAF1-FKBP_TTseq_contrast_cpm.txt.gz
SEM-SSRP1-FKBP_TTseq_featureCounts.txt.gz
SEM-SSRP1-FKBP_TTseq_contrast_cpm.txt.gz
SEM_NT-1_CapC_R1.fastq.gz
SEM_NT-1_CapC_R2.fastq.gz
SEM_NT-2_CapC_R1.fastq.gz
SEM_NT-2_CapC_R2.fastq.gz
SEM_NT-3_CapC_R1.fastq.gz
SEM_NT-3_CapC_R2.fastq.gz
SEM_MLLAF4-KD-1_CapC_R1.fastq.gz
SEM_MLLAF4-KD-1_CapC_R2.fastq.gz
SEM_MLLAF4-KD-2_CapC_R1.fastq.gz
SEM_MLLAF4-KD-2_CapC_R2.fastq.gz
SEM_MLLAF4-KD-3_CapC_R1.fastq.gz
SEM_MLLAF4-KD-3_CapC_R2.fastq.gz
SEM-PAF1-FKBP_DMSO-1_CapC_R1.fastq.gz
SEM-PAF1-FKBP_DMSO-1_CapC_R2.fastq.gz
SEM-PAF1-FKBP_DMSO-2_CapC_R1.fastq.gz
SEM-PAF1-FKBP_DMSO-2_CapC_R2.fastq.gz
SEM-PAF1-FKBP_DMSO-3_CapC_R1.fastq.gz
SEM-PAF1-FKBP_DMSO-3_CapC_R2.fastq.gz
SEM-PAF1-FKBP_dTag-1_CapC_R1.fastq.gz
SEM-PAF1-FKBP_dTag-1_CapC_R2.fastq.gz
SEM-PAF1-FKBP_dTag-2_CapC_R1.fastq.gz
SEM-PAF1-FKBP_dTag-2_CapC_R2.fastq.gz
SEM-PAF1-FKBP_dTag-3_CapC_R1.fastq.gz
SEM-PAF1-FKBP_dTag-3_CapC_R2.fastq.gz
SEM-SSRP1-FKBP_DMSO-1_CapC_R1.fastq.gz
SEM-SSRP1-FKBP_DMSO-1_CapC_R2.fastq.gz
SEM-SSRP1-FKBP_DMSO-2_CapC_R1.fastq.gz
SEM-SSRP1-FKBP_DMSO-2_CapC_R2.fastq.gz
SEM-SSRP1-FKBP_DMSO-3_CapC_R1.fastq.gz
SEM-SSRP1-FKBP_DMSO-3_CapC_R2.fastq.gz
SEM-SSRP1-FKBP_dTag-1_CapC_R1.fastq.gz
SEM-SSRP1-FKBP_dTag-1_CapC_R2.fastq.gz
SEM-SSRP1-FKBP_dTag-2_CapC_R1.fastq.gz
SEM-SSRP1-FKBP_dTag-2_CapC_R2.fastq.gz
SEM-SSRP1-FKBP_dTag-3_CapC_R1.fastq.gz
SEM-SSRP1-FKBP_dTag-3_CapC_R2.fastq.gz
SEM_MLLAF4-KD_CapC_combined.gfc.gz
SEM-PAF1-FKBP_CapC_combined.gfc.gz
SEM-SSRP1-FKBP_CapC_combined.gfc.gz
SEM-SIMM_RUNX1_R1.fastq.gz
SEM-SIMM_RUNX1_R2.fastq.gz
SEM-siMA6_MAZ_R1.fastq.gz
SEM-siMA6_MAZ_R2.fastq.gz
SEM-siMA6_MLL_R1.fastq.gz
SEM-siMA6_MLL_R2.fastq.gz
SEM-siMA6_MLL_input_R1.fastq.gz
SEM-siMA6_MLL_input_R2.fastq.gz
SEM-siMA6_RUNX1_R1.fastq.gz
SEM-siMA6_RUNX1_R2.fastq.gz
SEM-siMA6_prot_input_R1.fastq.gz
SEM-siMA6_prot_input_R2.fastq.gz
SEM-siMM_MAZ_R1.fastq.gz
SEM-siMM_MAZ_R2.fastq.gz
SEM-siMM_MLL_R1.fastq.gz
SEM-siMM_MLL_R2.fastq.gz
SEM-siMM_MLL_input_R1.fastq.gz
SEM-siMM_MLL_input_R2.fastq.gz
SEM-siMM_prot_input_R1.fastq.gz
SEM-siMM_prot_input_R2.fastq.gz
SEM-SIMM_RUNX1.bigWig
SEM-siMA6_RUNX1.bigWig
SEM-siMM_MAZ.bigWig
SEM-siMA6_MAZ.bigWig
SEM-siMM_MLL.bigWig
SEM-siMA6_MLL.bigWig
MM1S_PAF1_ChIP_R1.fastq.gz
MM1S_PAF1_ChIP_R2.fastq.gz
MM1S_Input_ChIP_R1.fastq.gz
MM1S_Input_ChIP_R2.fastq.gz
MM1S-PAF1-FKBP_H3K27ac_DMSO_ChIP_R1.fastq.gz
MM1S-PAF1-FKBP_H3K27ac_DMSO_ChIP_R2.fastq.gz
MM1S-PAF1-FKBP_Input_DMSO_ChIP_R1.fastq.gz

MM1S-PAF1-FKBP_Input_DMSO_ChIP_R2.fastq.gz
MM1S-PAF1-FKBP_H3K27ac_dTag_ChIP_R1.fastq.gz
MM1S-PAF1-FKBP_H3K27ac_dTag_ChIP_R2.fastq.gz
MM1S-PAF1-FKBP_Input_dTag_ChIP_R1.fastq.gz
MM1S-PAF1-FKBP_Input_dTag_ChIP_R2.fastq.gz
MM1S-PAF1-FKBP_DMSO_TTseq_R1.fastq.gz
MM1S-PAF1-FKBP_dTag_TTseq_R1.fastq.gz
Lib_1_4_15_R1.fastq.gz
Lib_1_4_25_R1.fastq.gz
Lib_5_4_30_R1.fastq.gz
Lib_5_4_40_R1.fastq.gz
Lib_6_4_20_R1.fastq.gz
Lib_6_5_20_R1.fastq.gz
Lib_1_4_15_R2.fastq.gz
Lib_1_4_25_R2.fastq.gz
Lib_5_4_30_R2.fastq.gz
Lib_5_4_40_R2.fastq.gz
Lib_6_4_20_R2.fastq.gz
Lib_6_5_20_R2.fastq.gz

Genome browser session
(e.g. UCSC)

No longer applicable

Methodology

Replicates

One replicate for ChIP-seq datasets and TT-seq in MM1.S cells. Three biological replicates for all Capture-C, MCC, Nascent RNA-seq and SEM TT-seq conditions.

Sequencing depth

chALL1_AF4_ChIP: total reads: 56016527, uniquely mapped reads: 55308120, read length: 40 bp, paired end
chALL1_Input_ChIP: total reads: 43014206, uniquely mapped reads: 42376639, read length: 40 bp, paired end
chALL1_MLL_ChIP: total reads: 71870452, uniquely mapped reads: 70979339, read length: 40 bp, paired end
chALL1_MLL_TOPM: total reads: 27018099, uniquely mapped reads: 26528278, read length: 40 bp, paired end
chALL2_H3K27ac_TOPM: total reads: 23041484, uniquely mapped reads: 22184636, read length: 40 bp, paired end
chALL2_H3K4me1_TOPM: total reads: 35161756, uniquely mapped reads: 34566197, read length: 40 bp, paired end
chALL2_H3K4me3_TOPM: total reads: 28864401, uniquely mapped reads: 27467304, read length: 40 bp, paired end
chALL2_H3K79me2_TOPM: total reads: 28224981, uniquely mapped reads: 27757612, read length: 40 bp, paired end
chALL3_AF4_ChIP: total reads: 124284126, uniquely mapped reads: 122064877, read length: 40 bp, paired end
chALL3_H3K27ac_TOPM: total reads: 17124065, uniquely mapped reads: 16783475, read length: 40 bp, paired end
chALL3_H3K27me3_TOPM: total reads: 13866474, uniquely mapped reads: 13562071, read length: 40 bp, paired end
chALL3_H3K4me1_TOPM: total reads: 10864143, uniquely mapped reads: 10647564, read length: 40 bp, paired end
chALL3_H3K4me3_TOPM: total reads: 15639803, uniquely mapped reads: 15271046, read length: 40 bp, paired end
chALL3_H3K79me2_TOPM: total reads: 20535319, uniquely mapped reads: 20141845, read length: 40 bp, paired end
chALL3_Input_ChIP: total reads: 11138417, uniquely mapped reads: 9660276, read length: 40 bp, paired end
chALL3_MLL_ChIP: total reads: 113430694, uniquely mapped reads: 110883002, read length: 40 bp, paired end
chALL3_MLL_TOPM: total reads: 15384725, uniquely mapped reads: 15003136, read length: 40 bp, paired end
chALL3_PAF1_TOPM: total reads: 14487615, uniquely mapped reads: 12748267, read length: 40 bp, paired end
chALL3_RUNX1_TOPM: total reads: 15342833, uniquely mapped reads: 14551822, read length: 40 bp, paired end
iALL2_H3K27ac_TOPM: total reads: 11620599, uniquely mapped reads: 11009123, read length: 40 bp, paired end
iALL2_H3K27me3_TOPM: total reads: 12982888, uniquely mapped reads: 12612160, read length: 40 bp, paired end
iALL2_H3K4me1_TOPM: total reads: 12556854, uniquely mapped reads: 12216722, read length: 40 bp, paired end
iALL2_H3K4me3_TOPM: total reads: 9376423, uniquely mapped reads: 8922505, read length: 40 bp, paired end
iALL2_H3K79me2_TOPM: total reads: 6629468, uniquely mapped reads: 6449666, read length: 40 bp, paired end
iALL2_Input_ChIP: total reads: 38212870, uniquely mapped reads: 37391743, read length: 40 bp, paired end
iALL2_MLL_ChIP: total reads: 113472670, uniquely mapped reads: 111367689, read length: 40 bp, paired end
iALL2_MLL_TOPM: total reads: 6526509, uniquely mapped reads: 6295169, read length: 40 bp, paired end
iALL2_RUNX1_TOPM: total reads: 1869659, uniquely mapped reads: 1819672, read length: 40 bp, paired end
RS411_ENL_HT-CM: total reads: 19528712, uniquely mapped reads: 18933122, read length: 40 bp, paired end
RS411_ENL_TOPM: total reads: 13614312, uniquely mapped reads: 12235985, read length: 40 bp, paired end
RS411_PAF1_HT-CM: total reads: 54162396, uniquely mapped reads: 52803733, read length: 40 bp, paired end
RS411_PAF1_TOPM: total reads: 20505986, uniquely mapped reads: 19503160, read length: 40 bp, paired end
SEM_H3K27ac-Input_NT_ChIP: total reads: 14049359, uniquely mapped reads: 13025556, read length: 40 bp, paired end
SEM_H3K27ac_NT_ChIP: total reads: 40342473, uniquely mapped reads: 36950507, read length: 40 bp, paired end
SEM_H3K79me3-Input_MLLAF4-KD_ChIP: total reads: 9955895, uniquely mapped reads: 8790090, read length: 40 bp, paired end
SEM_H3K79me3-Input_NT_ChIP: total reads: 25296316, uniquely mapped reads: 23090609, read length: 40 bp, paired end
SEM_H3K79me3_MLLAF4-KD_ChIP: total reads: 106840684, uniquely mapped reads: 90961176, read length: 40 bp, paired end
SEM_H3K79me3_NT_ChIP: total reads: 122066667, uniquely mapped reads: 103629292, read length: 40 bp, paired end
SEM-PAF1-FKBP_H3K27ac_DMSO_ChIP: total reads: 16359451, uniquely mapped reads: 15155923, read length: 40 bp, paired end
SEM-PAF1-FKBP_H3K27ac_dTag_ChIP: total reads: 28514951, uniquely mapped reads: 25212996, read length: 40 bp, paired end
SEM-PAF1-FKBP_Histone-Input_DMSO_ChIP: total reads: 4784030, uniquely mapped reads: 4514108, read length: 40 bp, paired end
SEM-PAF1-FKBP_Histone-Input_dTag_ChIP: total reads: 4541754, uniquely mapped reads: 4246050, read length: 40 bp, paired end
SEM-PAF1-FKBP_MLL_DMSO_ChIP: total reads: 28242584, uniquely mapped reads: 25976776, read length: 40 bp, paired end
SEM-PAF1-FKBP_MLL_dTag_ChIP: total reads: 71390562, uniquely mapped reads: 64102540, read length: 40 bp, paired end
SEM-PAF1-FKBP_MLL-Input_DMSO_ChIP: total reads: 9335758, uniquely mapped reads: 8504097, read length: 40 bp, paired end
SEM-PAF1-FKBP_MLL-Input_dTag_ChIP: total reads: 8141476, uniquely mapped reads: 7177895, read length: 40 bp, paired end
SEM-PAF1-FKBP_PAF1_DMSO_TOPM: total reads: 34447223, uniquely mapped reads: 30370167, read length: 40 bp, paired end
SEM-PAF1-FKBP_PAF1_dTag_TOPM: total reads: 44206477, uniquely mapped reads: 35659625, read length: 40 bp, paired end

SEM-PAF1-FKBP_PAF1-Input_DMSO_TOPM: total reads: 26866765, uniquely mapped reads: 25819971, read length: 40 bp, paired end
SEM-PAF1-FKBP_PAF1-Input_dTag_TOPM: total reads: 21466549, uniquely mapped reads: 20413844, read length: 40 bp, paired end
SEM_PAF1-Input_MLLAF4-KD_ChIP: total reads: 90605876, uniquely mapped reads: 84667335, read length: 40 bp, paired end
SEM_PAF1-Input_NT_ChIP: total reads: 119248457, uniquely mapped reads: 112735457, read length: 40 bp, paired end
SEM_PAF1_MLLAF4-KD_ChIP: total reads: 92558287, uniquely mapped reads: 88957910, read length: 40 bp, paired end
SEM_PAF1_NT_ChIP: total reads: 106008734, uniquely mapped reads: 100992550, read length: 40 bp, paired end
SEM_SSRP1_ChIP: total reads: 54938143, uniquely mapped reads: 53821715, read length: 40 bp, paired end
SEM-SSRP1-FKBP_H3K27ac_DMSO_ChIP: total reads: 33865794, uniquely mapped reads: 30833203, read length: 40 bp, paired end
SEM-SSRP1-FKBP_H3K27ac_dTag_ChIP: total reads: 26682492, uniquely mapped reads: 24075809, read length: 40 bp, paired end
SEM-SSRP1-FKBP_H3K79me3_DMSO_TOPM: total reads: 8510877, uniquely mapped reads: 7876421, read length: 40 bp, paired end
SEM-SSRP1-FKBP_H3K79me3_dTag_TOPM: total reads: 9628097, uniquely mapped reads: 8890957, read length: 40 bp, paired end
SEM-SSRP1-FKBP_Histone-Input_DMSO_ChIP: total reads: 6219112, uniquely mapped reads: 5802141, read length: 40 bp, paired end
SEM-SSRP1-FKBP_Histone-Input_dTag_ChIP: total reads: 7193665, uniquely mapped reads: 6713368, read length: 40 bp, paired end
SEM-SSRP1-FKBP_MLL_DMSO_ChIP: total reads: 83354909, uniquely mapped reads: 75306892, read length: 40 bp, paired end
SEM-SSRP1-FKBP_MLL_dTag_ChIP: total reads: 25659202, uniquely mapped reads: 23319757, read length: 40 bp, paired end
SEM-SSRP1-FKBP_MLL-Input_DMSO_ChIP: total reads: 16897189, uniquely mapped reads: 15078744, read length: 40 bp, paired end
SEM-SSRP1-FKBP_MLL-Input_dTag_ChIP: total reads: 9759909, uniquely mapped reads: 8712459, read length: 40 bp, paired end
SEM_SSRP1-Input_ChIP: total reads: 67019426, uniquely mapped reads: 65920447, read length: 40 bp, paired end
SIMM_RUNX1_ChIP: total reads: 21477718, uniquely mapped reads: 27494976, read length: 150 bp, paired end
siMA6_MAZ_ChIP: total reads: 17850242, uniquely mapped reads: 22146586, read length: 150 bp, paired end
siMA6_MLL_ChIP: total reads: 20384310, uniquely mapped reads: 27487754, read length: 150 bp, paired end
siMA6_MLL_input_ChIP: total reads: 15326296, uniquely mapped reads: 21033574, read length: 150 bp, paired end
siMA6_RUNX1_ChIP: total reads: 24072234, uniquely mapped reads: 29678496, read length: 150 bp, paired end
siMA6_prot_input_ChIP: total reads: 13931083, uniquely mapped reads: 17624034, read length: 150 bp, paired end
siMM_MAZ_ChIP: total reads: 27127576, uniquely mapped reads: 33488836, read length: 150 bp, paired end
siMM_MLL_ChIP: total reads: 21996804, uniquely mapped reads: 28678246, read length: 150 bp, paired end
siMM_MLL_input_ChIP: total reads: 16477212, uniquely mapped reads: 21368540, read length: 150 bp, paired end
siMM_prot_input_ChIP: total reads: 18631846, uniquely mapped reads: 26023236, read length: 150 bp, paired end
MM1S_PAF1_ChIP: total reads: 60636385 uniquely mapped reads: 58135650 read length: 40 bp, paired end
MM1S_Input_ChIP: total reads: 9737332 uniquely mapped reads: 5773646 read length: 40 bp, paired end
MM1S-PAF1-FKBP_H3K27ac_DMSO_ChIP: total reads: 32769406 uniquely mapped reads: 32769406 read length: 150 bp, paired end
MM1S-PAF1-FKBP_H3K27ac_dTag_ChIP: total reads: 17835764 uniquely mapped reads: 11871638 read length: 150 bp, paired end
MM1S-PAF1-FKBP_Input_DMSO_ChIP: total reads: 1559856 uniquely mapped reads: 786076 read length: 150 bp, paired end
MM1S-PAF1-FKBP_Input_dTag_ChIP: total reads: 1934747 uniquely mapped reads: 1002064 read length: 150 bp, paired end

Antibodies

anti-AF4-C: Abcam ab31812 lot GR33890-1
anti-ENL: Bethyl A302-268A lot 1
anti-H3K27ac: Diagenode C15410196 lot A1723-0041D
anti-H3K27me3: Sigma-Aldrich 07-449 lot 2318778
anti-H3K4me1: Diagenode C15410194 lot A1862D
anti-H3K4me3: Diagenode C15410194 lot A1052D
anti-H3K79me2: Sigma-Aldrich 04-835 lot NG1800794
anti-H3K79me3: Diagenode C15410068 lot A246-0040
anti-MAZ: Bethyl A301-652A lot 1
anti-MLL-N: Bethyl BA300-086A lot 6
anti-PAF1: Bethyl A300-172A lot 3
anti-RUNX1: Cell Signaling 4334 lot 3
anti-SSRP1: Bethyl A303-067A lot 1

Peak calling parameters

Read Mapping: ChIP-seq: bowtie2 with default parameters
ATAC-seq: bowtie2 -X 2000
Peak calling:
Homer:
findPeaks, with the input track provided for background correction, using the -style histone or -style factor options to call peaks in histone modification or transcription factor/ATAC datasets, respectively.
LanceOtron:
lanceotron callpeaks -c 0.5

Data quality

Reads were filtered to remove PCR duplicates. Homer called peaks were analyzed as described on homer.ucsd.edu and compared to input track, with a threshold of FDR < 0.001 applied. LanceOtron called peaks were filtered for peaks with a peak score > 0.5.

Software

Quality control of FASTQ reads was performed using FastQC v0.12.1, adapters and poor quality bases were removed using trim_galore v0.6.10. Samples were aligned to the hg19 genome assembly using bowtie2 v2.5.1. PCR duplicates were removed using picard MarkDuplicates v3.0.0. Problematic genomic regions present in the ENCODE Blacklist (https://doi.org/10.1038/s41598-019-45839-z) were removed from the aligned files and further QC of the aligned files was performed using samtools v1.17. BigWigs were generated using the deepTools (v3.5.1) bamCoverage command 128, with the flags –extendReads –normalizeUsing RPKM. As many of the factors that were immunoprecipitated have a mix of both sharp and broad modalities, we used either HOMER v4.11 or the deep learning based peak caller LanceOtron v1.0.8 (with a peak score cut-off value of 0.5) to call peaks. UCSC data hub generation for the BigWig and peak files was performed using a custom script.
